# Supplementary material for: Visible-light-excited robust room-temperature phosphorescence of dimeric single-component luminophores in the amorphous state
Source: Nat Commun. 2024 Apr 27;15:3598. doi: 10.1038/s41467-024-47937-7 (PMC11055858; doi:10.1038/s41467-024-47937-7)
Supplement: Supplementary file 1 — Supplementary Infomation [file 41467_2024_47937_MOESM1_ESM.pdf]

Supplementary information

Visible-light-excited robust room-temperature phosphorescence of dimeric single-component luminophores in the amorphous state

Guo et al.

Supplementary Note 1. Additional characterization of TpPX

Phenyl(triphenylen-2-yl)methanone (TpP).

^{1}H NMR (400 MHz, Chloroform-*d*) δ ppm: 9.09 (d, *J* = 1.8 Hz, 1H), 8.76 (d, *J* = 8.6 Hz, 1H), 8.73 – 8.66 (m, 3H), 8.65 – 8.59 (m, 1H), 8.05 (dd, *J* = 8.5, 1.7 Hz, 1H), 7.96 – 7.90 (m, 2H), 7.80 – 7.68 (m, 3H), 7.66 (d, *J* = 1.9 Hz, 2H); ^{13}C NMR (151 MHz, Chloroform-*d*) δ ppm: 196.66, 137.88, 135.77, 132.93, 132.55, 130.73, 130.21, 130.03, 129.51, 129.44, 129.02, 128.46, 128.39, 128.08, 127.85, 127.61, 127.55, 126.05, 124.04, 123.53, 123.50, 123.44, 123.41; HMRS (TOF MS ES+): *m*/*z* calculated for $C_{25}H_{17}O$ $[M+H]^+$ 332.1274, found 332.1276.

(4-fluorophenyl)(triphenylen-2-yl)methanone (TpPF).

^{1}H NMR (400 MHz, Dichloromethane-d_2) δ ppm: 9.12 (d, *J* = 1.7 Hz, 1H), 8.82 (d, *J* = 8.5 Hz, 1H), 8.80 – 8.71 (m, 3H), 8.71 – 8.65 (m, 1H), 8.09 (dd, *J* = 8.5, 1.7 Hz, 1H), 8.06 – 7.96 (m, 2H), 7.84 – 7.68 (m, 4H), 7.35 – 7.23 (m, 2H); ^{13}C NMR (151 MHz, Chloroform-*d*) δ ppm: 195.15, 166.33, 164.64, 135.65, 134.12, 134.10, 132.96, 132.81, 132.75, 130.73, 130.05, 129.47, 129.42, 128.95, 128.43, 127.90, 127.85, 127.63, 125.84, 124.01, 123.50, 123.45, 123.43, 115.70, 115.56; HMRS (TOF MS ES+): *m*/*z* calculated for $C_{25}H_{16}FO$ $[M+H]^+$ 351.1180, found 351.1180.

(4-methoxyphenyl)(triphenylen-2-yl)methanone (TpPBr).

^{1}H NMR (400 MHz, Chloroform-*d*) δ ppm: 9.09 (d, *J* = 1.8 Hz, 1H), 8.76 (d, *J* = 8.6 Hz, 1H), 8.73 – 8.65 (m, 3H), 8.65 – 8.59 (m, 1H), 8.05 (dd, *J* = 8.6, 1.8 Hz, 1H), 7.85 – 7.78 (m, 2H), 7.78 – 7.66 (m, 6H); ^{13}C NMR (151 MHz, Chloroform-*d*) δ ppm: 193.53, 136.74, 134.98, 133.28, 131.93, 131.84, 130.92, 130.19, 129.64, 129.53, 129.07, 128.65, 128.10, 128.00, 127.82, 127.78, 127.73, 126.12, 124.20, 123.74, 123.66, 123.62, 123.59; HMRS (TOF MS ES+): *m*/*z* calculated for $C_{25}H_{16}BrO$ $[M+H]^+$ 411.0379, found 411.0381.

(4-iodophenyl)(triphenylen-2-yl)methanone (TpPI).

^{1}H NMR (400 MHz, Chloroform-*d*) δ ppm: 9.09 (d, *J* = 1.8 Hz, 1H), 8.76 (d, *J* = 8.6 Hz, 1H), 8.73 – 8.66 (m, 3H), 8.65 – 8.59 (m, 1H), 8.05 (dd, *J* = 8.5, 1.7 Hz, 1H), 7.96 – 7.90 (m, 2H), 7.80 – 7.68 (m, 3H), 7.66 (d, *J* = 1.9 Hz, 2H); ^{13}C NMR (151 MHz, Chloroform-*d*) δ ppm: 196.66, 137.88, 135.77, 132.93, 132.55, 130.73, 130.21, 130.03, 129.51, 129.44, 129.02, 128.46, 128.39, 128.08, 127.85, 127.61, 127.55, 126.05, 124.04, 123.53, 123.50, 123.44, 123.41; HMRS (TOF MS ES+): *m*/*z* calculated $C_{25}H_{16}IO$ $[M+H]^+$ 459.0240, found 459.0242.

p-tolyl(triphenylen-2-yl)methanone (TpPMe).

^{1}H NMR (400 MHz, Chloroform-*d*) δ ppm: 9.10 (d, *J* = 1.7 Hz, 1H), 8.73 (d, *J* = 8.5 Hz, 1H), 8.71 – 8.65 (m, 3H), 8.65 – 8.60 (m, 1H), 8.07 (dd, *J* = 8.5, 1.7 Hz, 1H), 7.85 (d, *J* = 8.2 Hz, 2H), 7.76 – 7.63 (m, 4H), 7.35 (d, *J* = 7.8 Hz, 2H), 2.50 (s, 3H); ^{13}C NMR (151 MHz, Chloroform-*d*) δ ppm: 196.02, 143.51, 136.28, 135.30, 132.88, 130.81, 130.58, 130.16, 129.69, 129.52, 129.29, 129.21, 128.44, 128.16, 127.92, 127.71, 127.65, 126.02, 124.13, 123.67, 123.61, 123.53, 123.51, 21.87; HMRS (TOF MS ES+): *m*/*z* calculated for $C_{26}H_{19}O$ $[M+H]^+$ 347.1430, found 347.1432.

(4-methoxyphenyl)(triphenylen-2-yl)methanone (TpPOMe).

^{1}H NMR (400 MHz, Chloroform-*d*) δ ppm: 9.07 (d, *J* = 1.7 Hz, 1H), 8.75 (d, *J* = 8.6 Hz, 1H), 8.73 – 8.66 (m, 3H), 8.66 – 8.62 (m, 1H), 8.05 (dd, *J* = 8.4, 1.7 Hz, 1H), 7.95 (d, *J* = 8.9 Hz, 2H), 7.77 – 7.64 (m, 4H), 7.04 (d, *J* = 8.9 Hz, 2H), 3.93 (s, 3H); ^{13}C NMR (151 MHz, Chloroform-*d*) δ ppm: 197.30, 162.78, 136.67, 132.84, 132.70, 131.14, 130.60, 130.18, 129.71, 129.53, 129.26, 128.40, 128.07, 127.93, 127.72, 127.66, 125.34, 124.11, 123.68, 123.63, 123.55, 123.50, 114.97, 55.31; HMRS (TOF MS ES+): *m*/*z* calculated for $C_{26}H_{19}O_2$ $[M+H]^+$ 363.1380, found 363.1383.

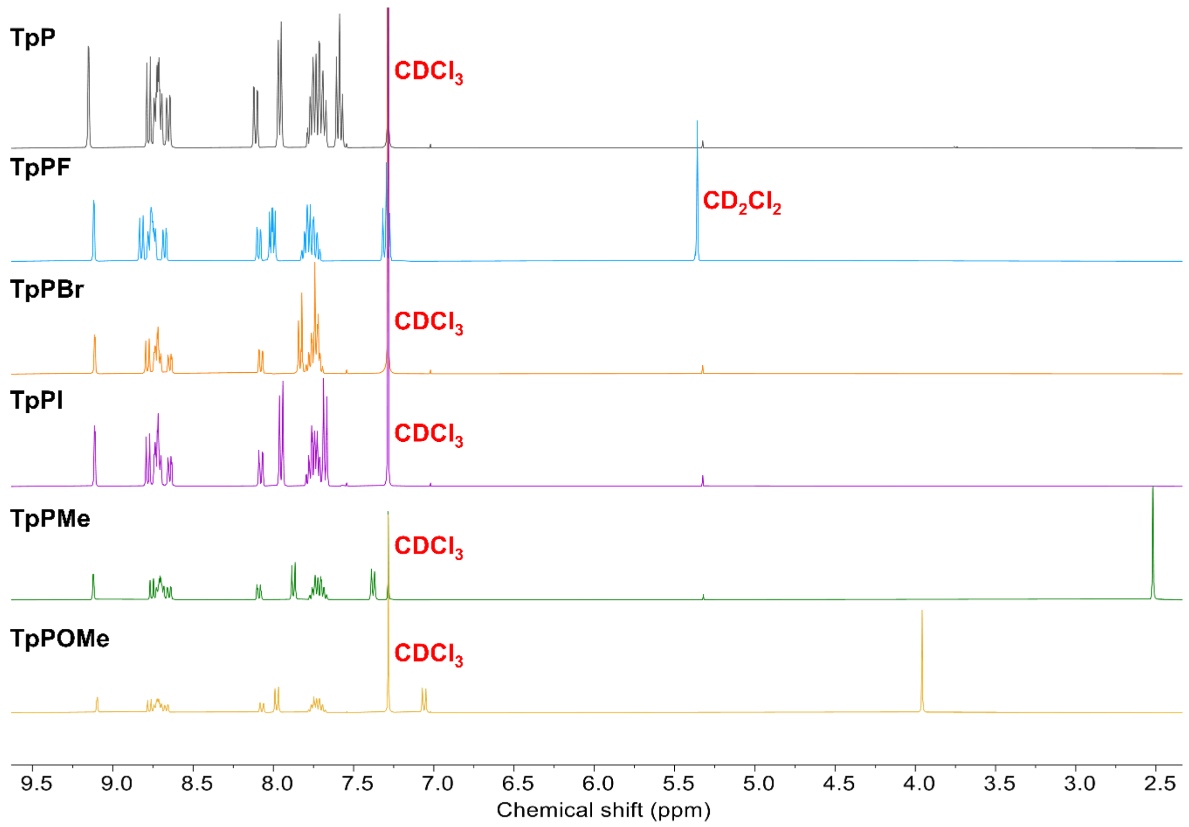

Supplementary Figure 1. ^{1}H NMR spectra of TpP (black line), TpPF (blue line), TpPBr (orange line), TpPI (purple line), TpPMe (green line) and TpPOMe (yellow line). The solvent was deuterated chloroform ($CDCl_3$) or deuterated dichloromethane (Dichloromethane-d_2), and the internal standard was tetramethylsilane.

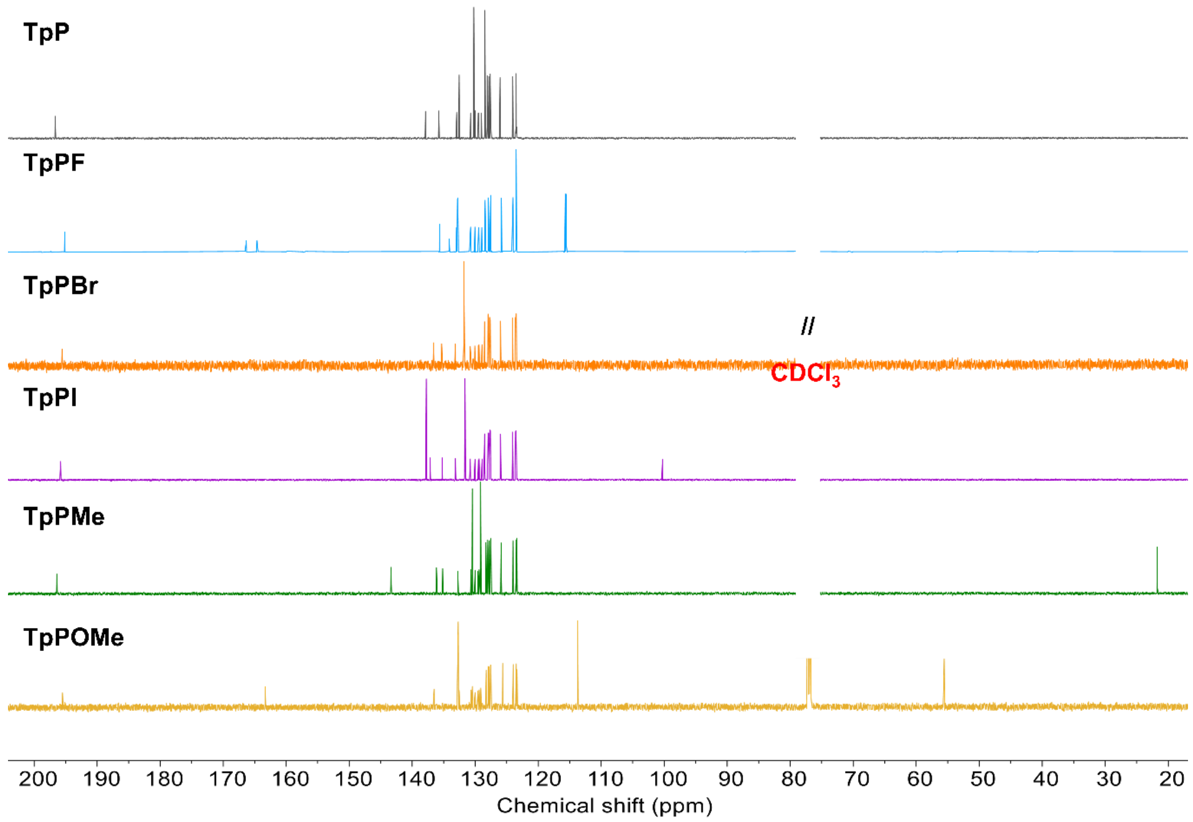

Supplementary Figure 2. ^{13}C NMR spectra of TpP (black line), TpPF (blue line), TpPBr (orange line), TpPI (purple line), TpPMe

(green line) and TpPOMe (yellow line). The solvent was deuterated chloroform ($CDCl_3$) and the internal standard was tetramethylsilane.

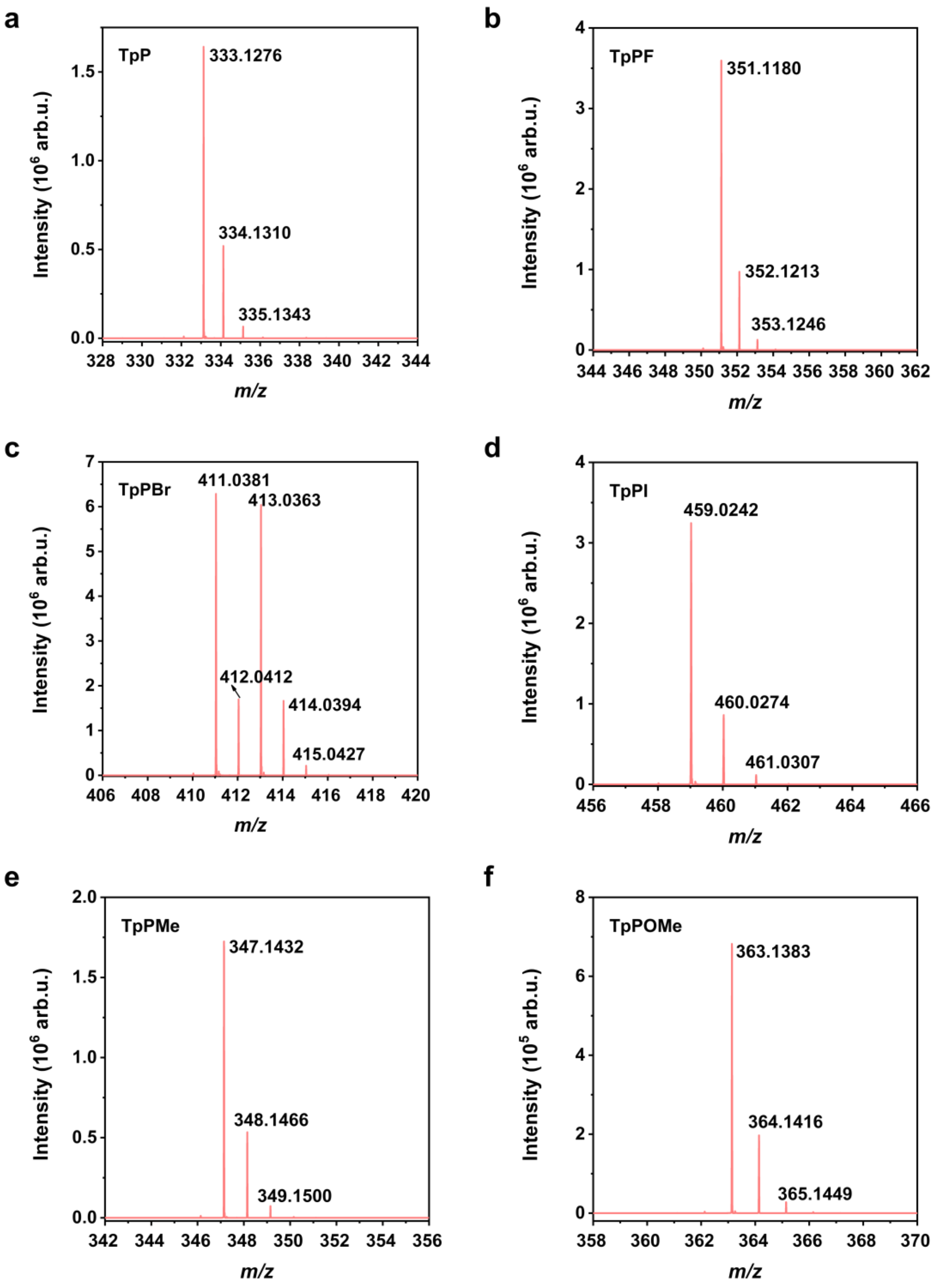

Supplementary Figure 3. MALDI-TOF MS of TpP (**a**), TpPF (**b**), TpPBr (**c**), TpPI (**d**), TpPMe (**e**) and TpPOMe (**f**).

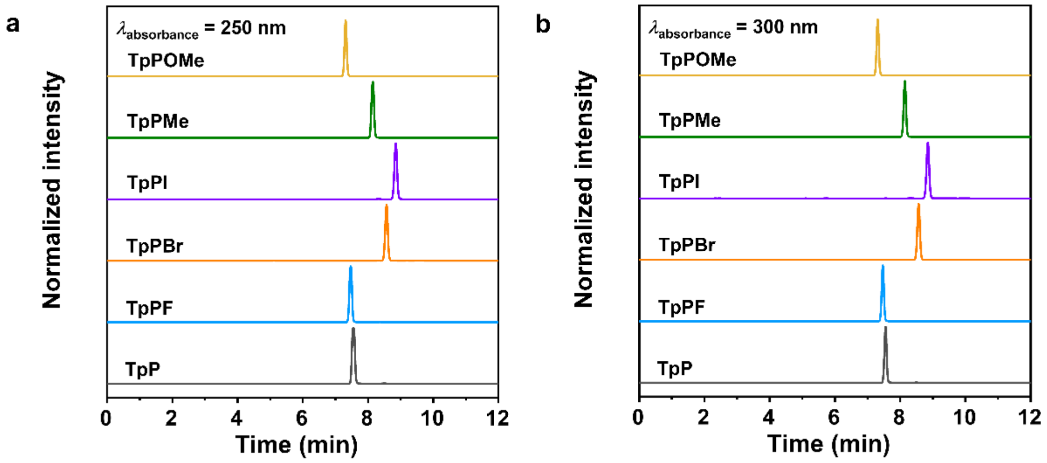

Supplementary Figure 4. HPLC of TpPX at an asborbance wavelength of 250 nm (**a**) and 300 nm (**b**).

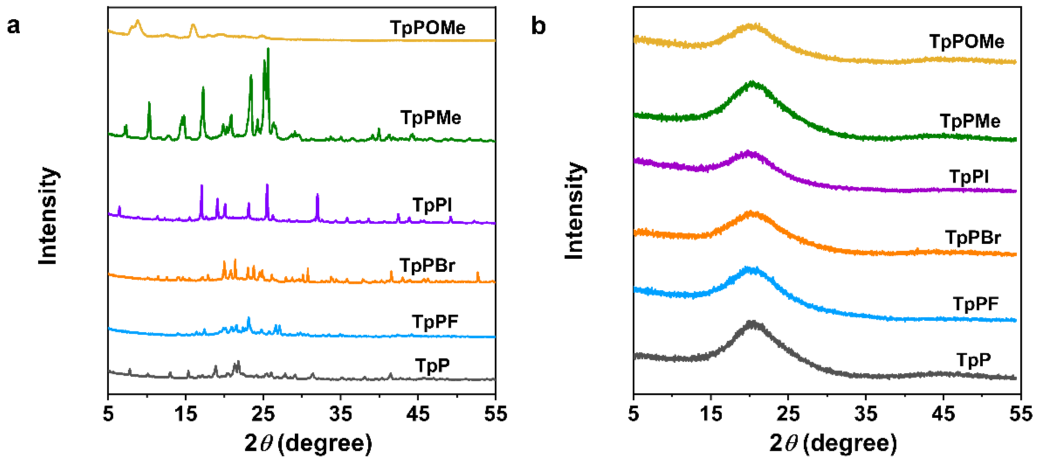

Supplementary Figure 5. XRD of pristine (**a**) and amorphous (**b**) TpPX powder.

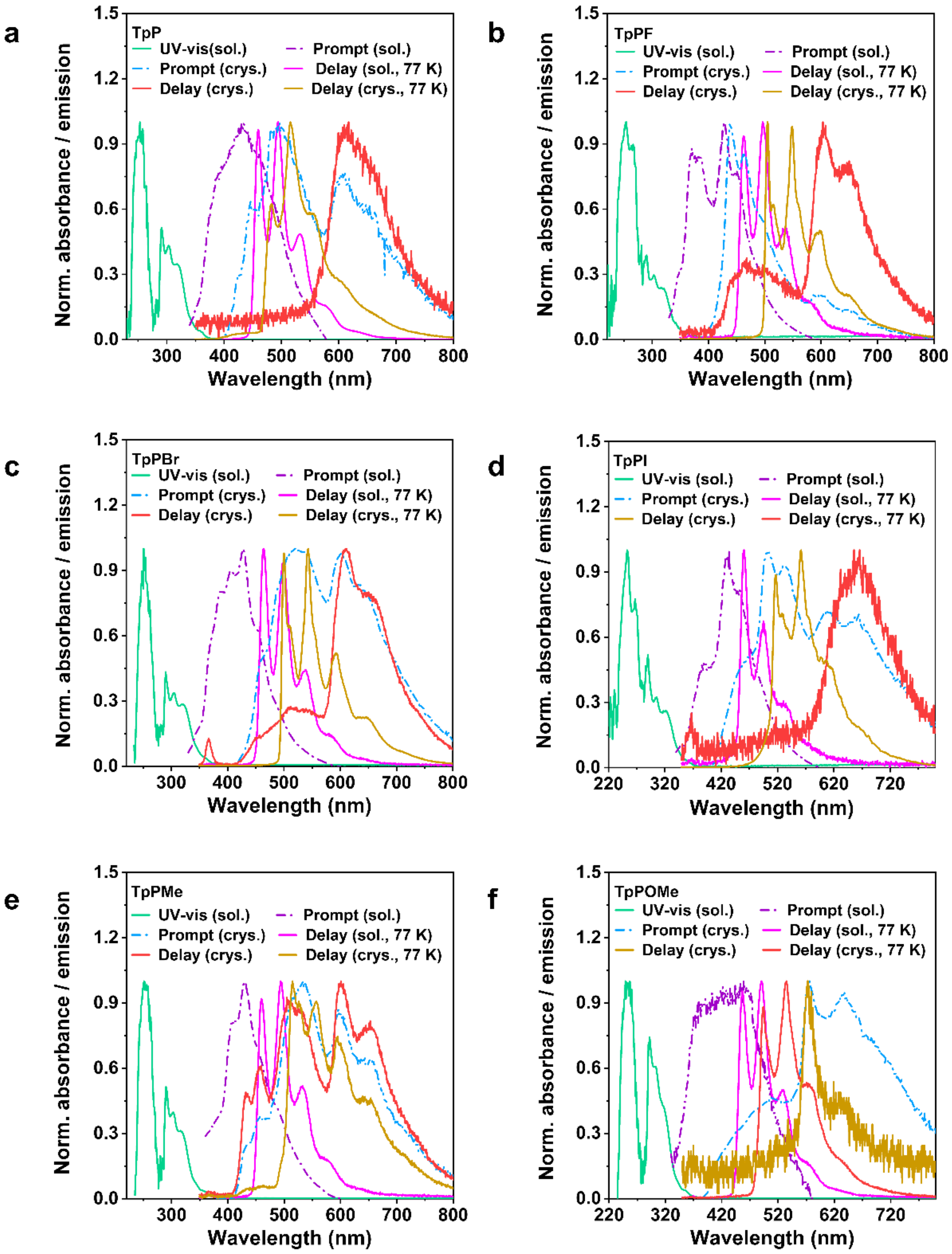

Supplementary Figure 6. Photophysical properties of TpPX. Normalized UV-vis absorption (green solid line), prompt luminescence spectra (purple dashed line), and delayed luminescence spectra (pink solid line) of TpP (**a**), TpPF (**b**), TpPBr (**c**), TpPI (**d**), TpPMe (**e**), and TpPOMe (**f**) in 2-Methyltetrahydrofuran (UV-vis: 5 × 10^{-5} M, 298 K, in air; Prompt: TpP and TpPOMe @ 10^{-3} M, other compound @ 5 × 10^{-5} M, 298 K, in air, λ_{ex} = 310 nm; Delayed: 1 × 10^{-5} M; 77 K, in air, λ_{ex} = 365 nm, delayed 8 ms). Prompt (blue dashed line) and delayed (RT: red solid line; 77 K: yellow solid line) luminescence spectra of TpP (**a**), TpPF (**b**), TpPBr (**c**), TpPI (**d**), TpPMe (**e**), and TpPOMe (**f**) in crystalline state (Prompt: 298 K, in air, λ_{ex} = 365 nm; Delayed: 298 K & 77 K, in air, λ_{ex} = 365 nm, delayed 8 ms). Sol., solution; Crys., crystalline.

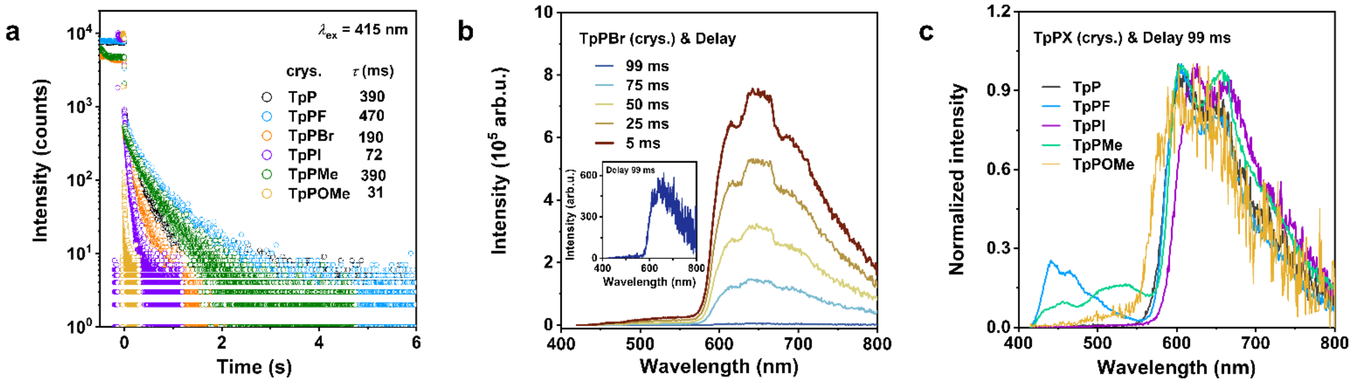

Supplementary Figure 7. a, PL intensity decay profiles of crystalline TpPX powder at their corresponding peaks (ca. 600 - 630 nm) (298 K, air, λ_{ex} = 415 nm). **b**, Delayed luminescence spectra of TpPBr powder with different delay times (298 K, air, λ_{ex} = 405 nm). Test slits were not adjusted. **c**, Delayed luminescence spectra of other TpPX powder with a delay time of 99 ms (298 K, air, λ_{ex} = 405 nm).

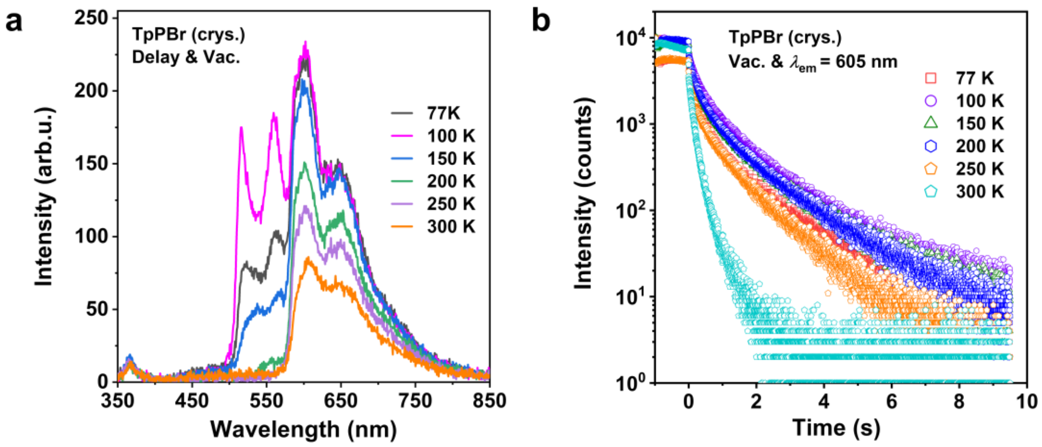

Supplementary Figure 8. a, Delayed luminescence spectra of TpPBr measured at temperatures from 77 to 300 K in a vacuum (λ_{ex} = 365 nm). **b**, PL intensity decay profiles of crystalline TpPBr powder at peak of 605 nm in a vacuum (λ_{ex} = 340 nm).

Supplementary Note 2. The emission at 515 and 560 nm might be from the high-lying triplet states of aggregated states (T_2^{dimer}), due to the suppression of internal conversion (IC) and non-radiative transitions at low temperatures. When the temperature increased from 77 K to 100 K, the phosphorescence enhancement of T_2^{dimer} was mainly relative to two reasons. One reason was that the thermal equilibrium of excitons converting from T_2^{dimer} to T_1^{dimer} states, as their moderate energy gap. Another reason was the bigger phosphorescence rate (k_p) of T_2^{dimer}, which could suppress the IC transition from T_2^{dimer} to T_1^{dimer} and subsequently promote the emission of T_2^{dimer}. As the temperature further increased, the non-radiative transitions of T_2^{dimer} excitons were promoted, and its emission was weakened (reference such as *Mol. Phys.* **27**, 969-979 (1974); *J. Phys. Chem.* **91**, 819 (1987); *Chem. Rev.* **112**, 4541-4568 (2012)).

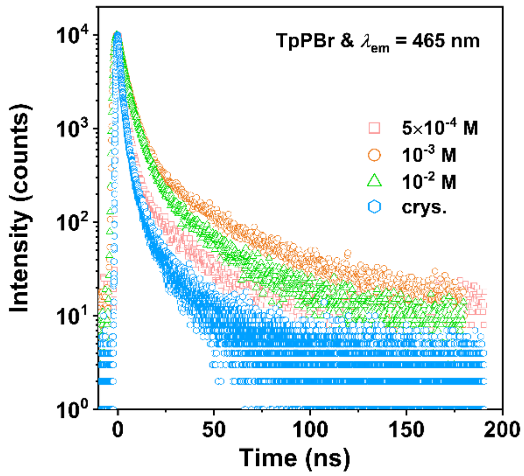

Supplementary Figure 9. Fluorescence intensity decay profiles of TpPBr in 1,4-dioxane solutions with incremental concentrations or its crystalline powder (298 K, air, λ_{ex} = 405 nm).

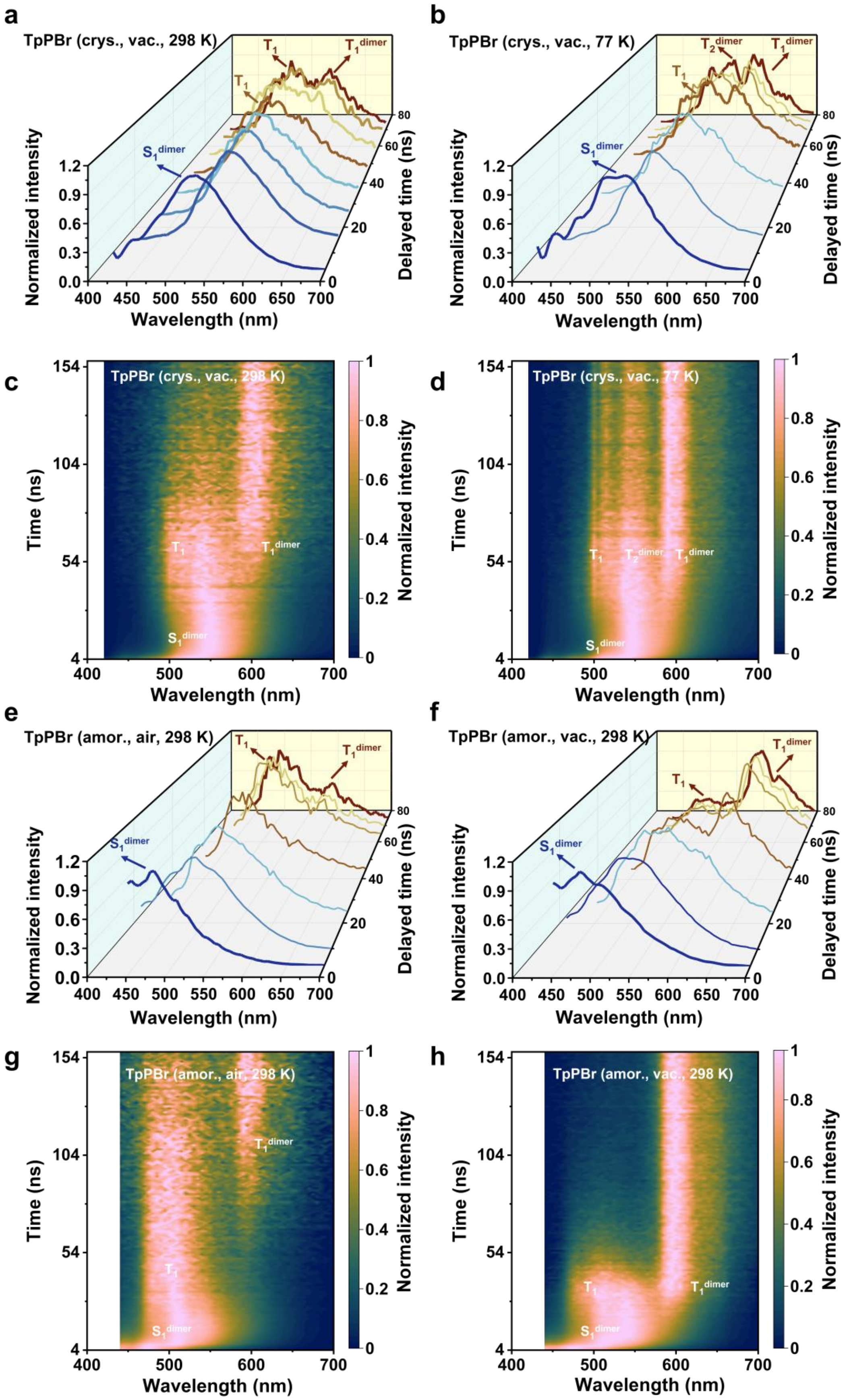

Supplementary Figure 10. Normalized time-resolved emission spectroscopy (TRES) of TpPBr in crystalline or amorphous state in air/in a vacuum at 298/77 K at nanosecond scale (λ_{ex} = 405 nm, crys. & vac. & 298 K for **a** and **c**, crys. & vac. & 77 K

for **b** and **d**, amor. & air & 298 K for **e** and **g**, amor. & vac. & 298 K for **f** and **h**). Crys., crystalline.; Amor., amorphous; Vac., vacuum.

Supplementary Note 3. In the time-resolved spectral decay of TpPBr, the significant emission peaks (560 nm) for T_2^{dimer} appeared simultaneously and decayed along with the T_1^{dimer} peak at ≈600 nm. However, the decay of T_2^{dimer} emission was much faster than T_1^{dimer}, indicating its short lifetime and subsequent faster radiation with bigger rate (k_{p}).

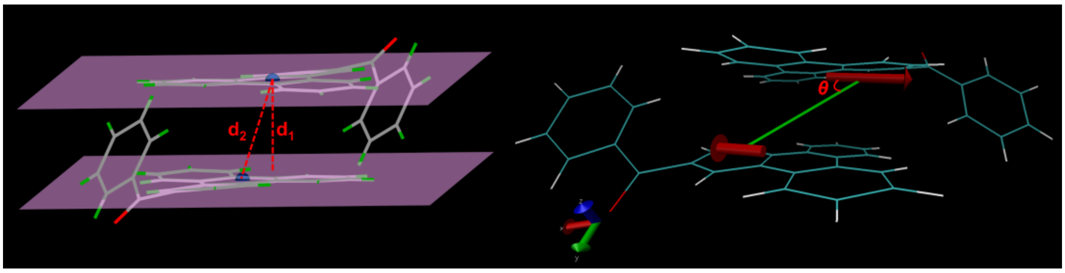

Supplementary Figure 11. Schematic representation of aggregation models. The blue sphere and purple plane are the centroid and plane of the triphenylene core, respectively. And d_1 represent vertical distance between two adjacent triphenylene planes while d_2 was the centroid distance of two adjacent triphenylene. The red arrow describes the transition dipole moment of the monomer in the dimer. The green line represents the centroid of the monomer in the dimer. And the angle between the transition dipoles and the interconnected axis is indicated by θ. The transition dipole moment is calculated using the Gaussian 09W package.

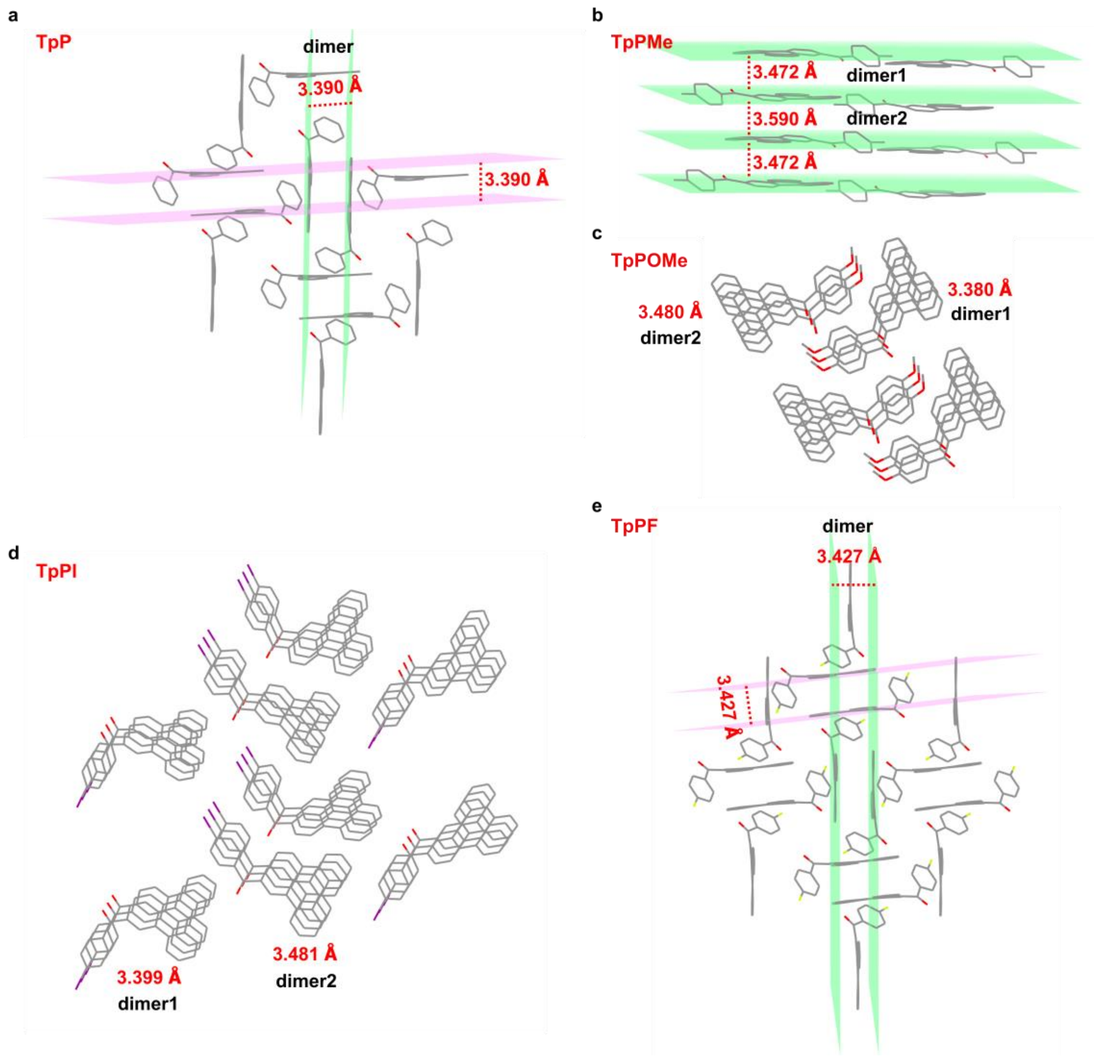

Supplementary Figure 12. Crystal structures of TpP (**a**), TpPMe (**b**), TpPOMe (**c**), TpPI (**d**), TpPF (**e**). Distance represents the vertical distance between two adjacent triphenylene planes.

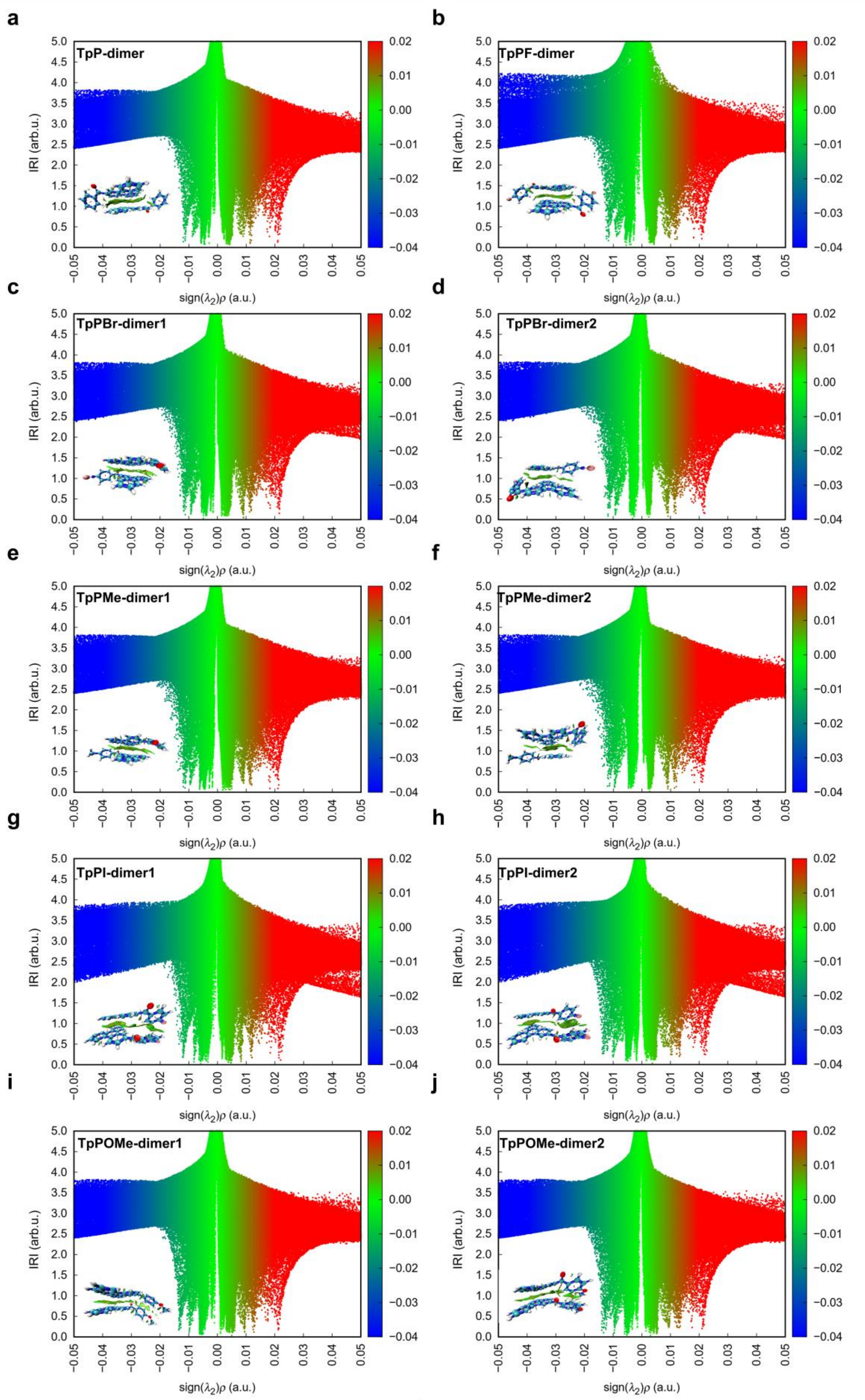

Supplementary Figure 13. Scatter plots and dstribution regions (inset) of intramolecular/intermolecular interactions in TpP-

dimer (**a**), TpPF-dimer (**b**), TpPBr-dimer1 (**c**), TpPBr-dimer2 (**d**), TpPMe-dimer1 (**e**), TpPBr-dimer2 (**f**), TpPI-dimer1 (**g**), TpPI-dimer2 (**h**), TpPOMe-dimer1 (**i**), TpPOMe-dimer2 (**j**), calculated and visualised based on Gaussian, Multiwfn and VMD[1,2]. The sign(λ_2)*p* function can be coloured on IRI isosurfaces to illustrate the nature of interaction regions revealed by IRI. A decrease in sign(λ_2)*p* is indicated by the colour bar changing from red to green and then to blue. When sign(λ_2)*p* is larger, that is, the isosurface of IRI becomes redder, it indicates a notable repulsion, such as a strong steric hindrance. When sign(λ_2)*p* decreases and the isosurface is significantly blue, it implies a significant attractive effect, such as hydrogen bonds and halogen bonds of general strength. When the isosurface is basically green, it indicates a noticeable interaction, such as vDW interaction or a weak hydrogen bond.

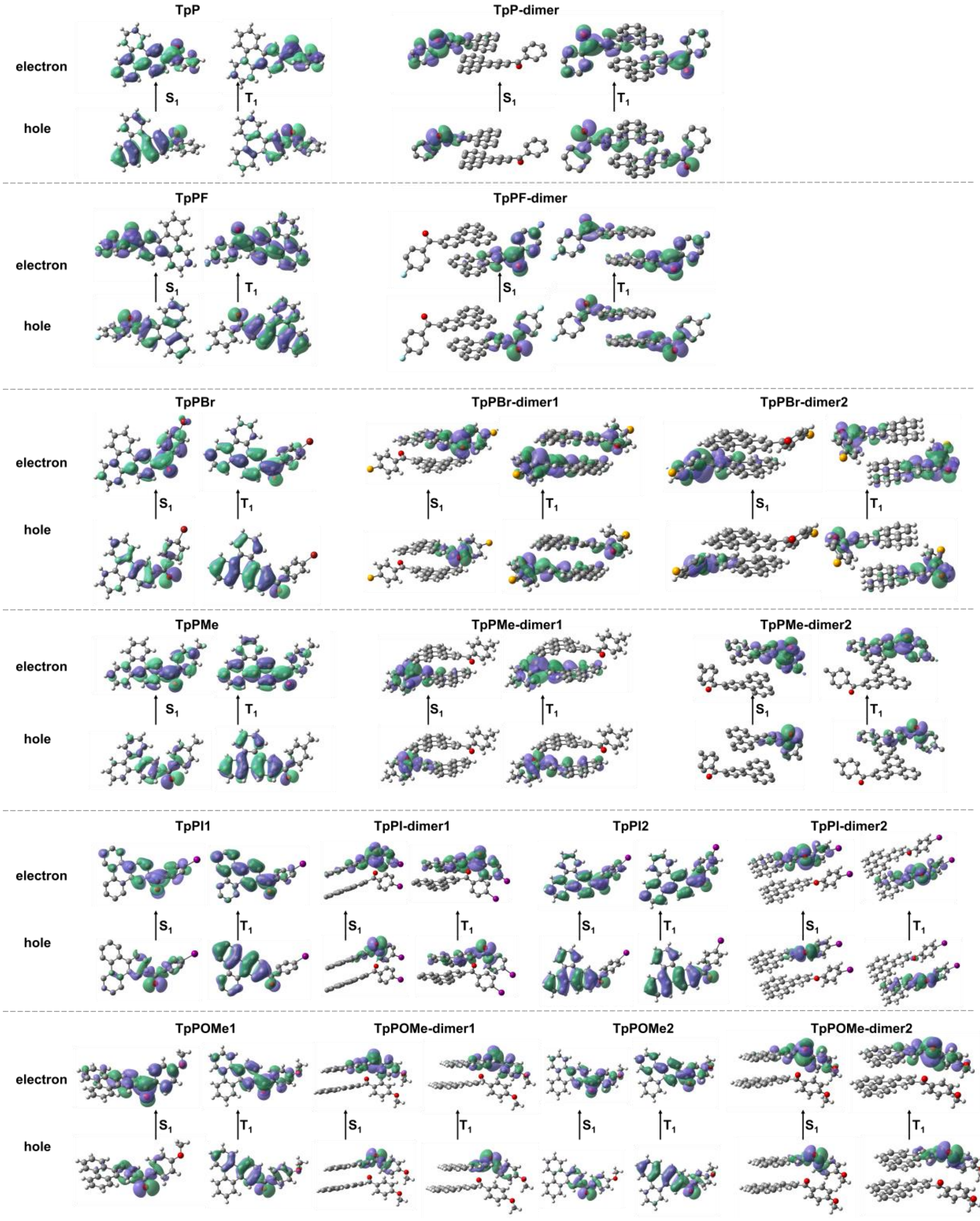

Supplementary Figure 14. Natural transition orbitals of S_1 and T_1 in TpPX monomer and its dimers. The purple and green colors represent the positive and negative signs of the molecular orbital wave function, respectively. The isovalue surface is 0.02 e $Bohr^{-3}$. The arrow indicates the excitation process of S_0->S_1 or S_0->T_1.

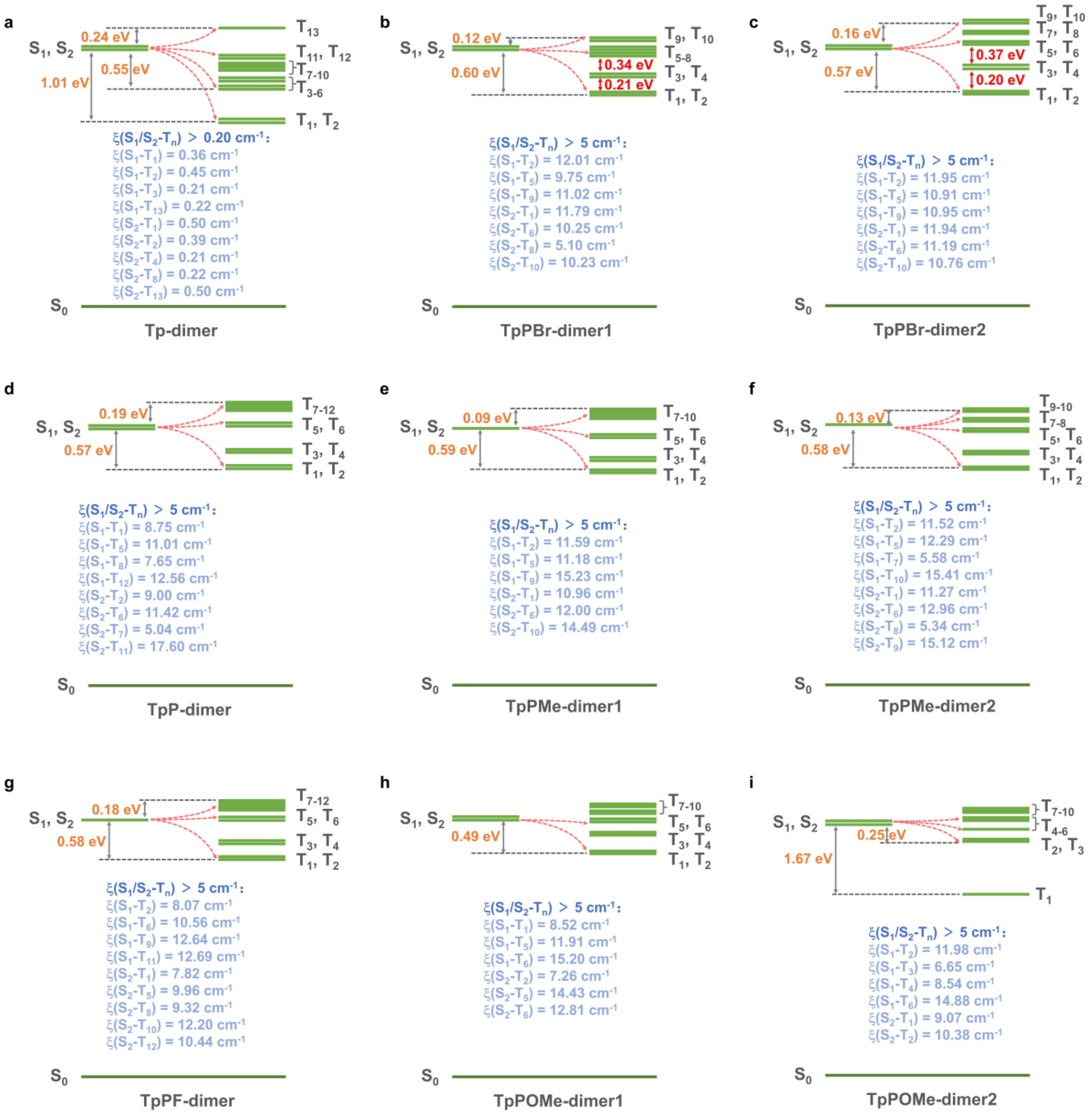

Supplementary Figure 15. Theoretical calculated spin-orbit coupling (SOC) matrix element of Tp (**a**), TpPBr-dimer1 (**b**), TpPBr-dimer2 (**c**), TpPdimer (**d**), TpPMe-dimer1 (**e**), TpPMe-dimer2 (**f**), TpPF-dimer (**g**), TpPOMe-dimer1 (**h**) and TpPOMe-dimer2 (**i**) from their single crystals.

Supplementary Note 4. Take TpPBr (b) as an example. The SOC matrix elements were calculated and showed that the energy gaps of T_2-T_3 and T_4-T_5 were 0.21 eV and 0.34 eV, respectively, which were two of the largest among the energy gaps between T_ns. The total spin-orbit coupling matrix elements of S_1/S_2 and T_3-T_{10} were up to 55.5 cm^{-1}, indicating that the transition from S_1/S_2 to T_n (T_3-T_{10}) was possible.

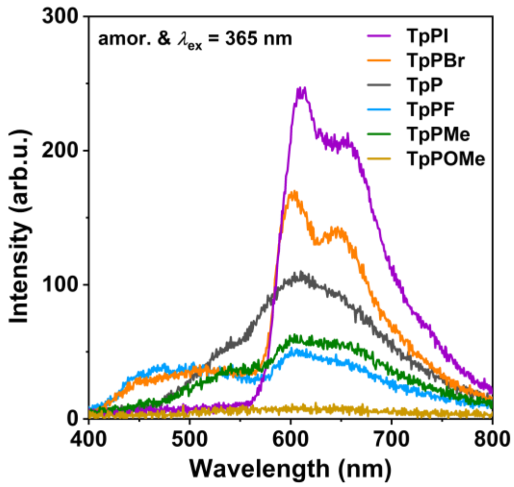

Supplementary Figure 16. Delayed luminescence spectra of amorphous TpPX powder under the excitation at 365 nm (298 K, in air, delayed 8 ms). Amor., amorphous.

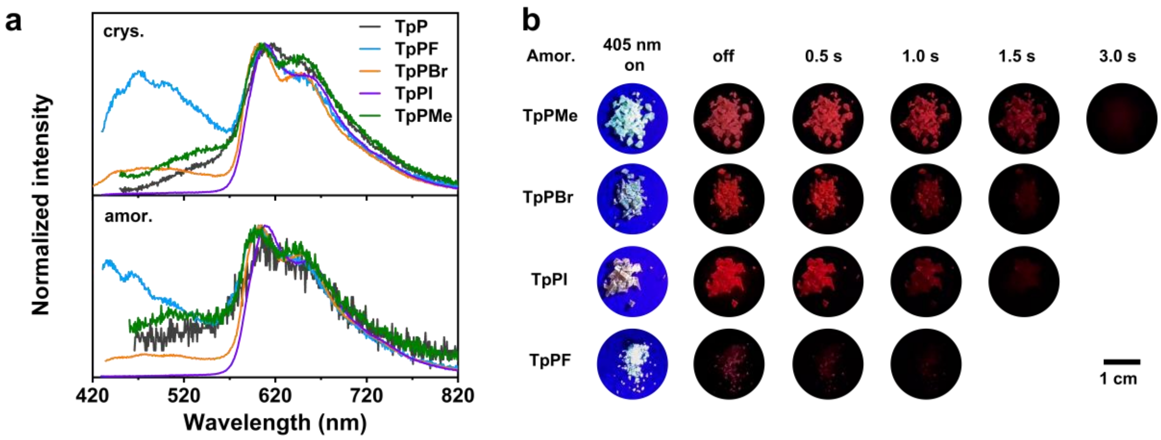

Supplementary Figure 17. a, Delayed luminescence spectra of crystalline and amorphous TpPX sample (298 K, in air, λ_{ex} = 405 nm). **b**, Luminescent photographs of amorphous TpPX powder captured using an iphone12 camera before and after turning off the excited light (298 K, in air, λ_{ex} = 405 nm). Crys., crystalline; Amor., amorphous.

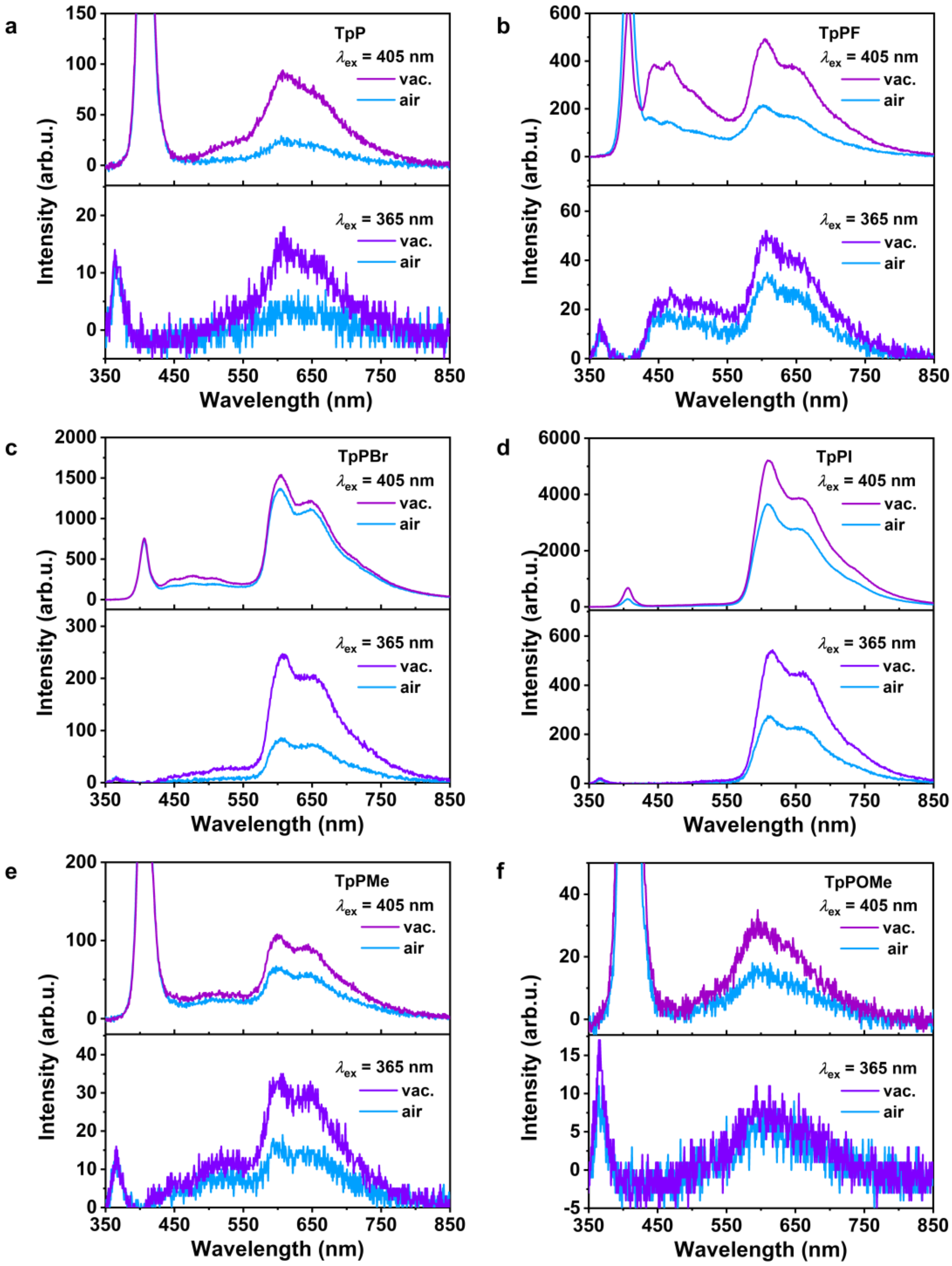

Supplementary Figure 18. Delayed luminescence spectra of amorphous TpP (**a**), TpPF (**b**), TpPBr (**c**), TpPI (**d**), TpPMe (**e**) and TpPOMe (**f**) film in a vacuum (purple solid line) or in air (blue solid line) at different excitation wavelength (298 K, delayed 8 ms). Vac., vacuum.

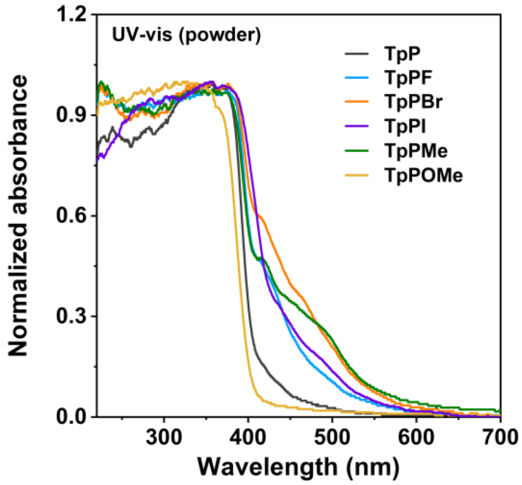

Supplementary Figure 19. UV-Vis spectra of TpPX powder.

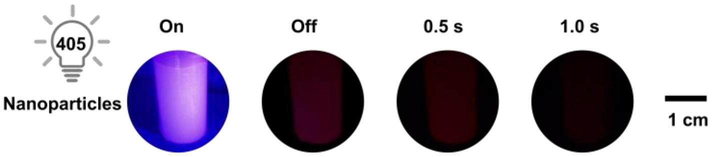

Supplementary Figure 20. Luminescent photographs of TpPBr@F127 nanoparticles in H_2O (2.44 mM) captured using an iphone12 camera after turning off the excited light (λ_{ex} = 405 nm).

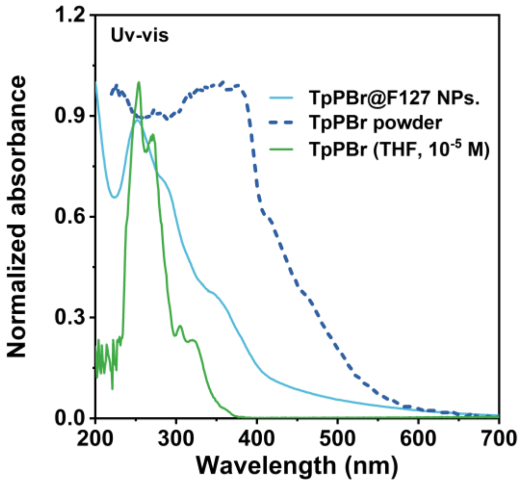

Supplementary Figure 21. Normalized UV-vis absorption of TpPBr in THF (10^{-5} M), the aqueous solution of TpPBr@F127 nanoparticles, and TpPBr powder. NPs., nanoparticles.

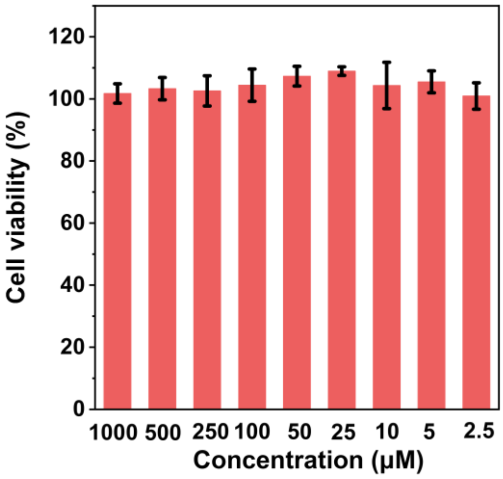

Supplementary Figure 22. Cell viabilities of RM-1 cells after co-incubation with TpPBr nanoparticles in different concentration. The cell viability is the ratio of the MTT absorbance of each well to that of the control well. Graph represents the mean values ±SD of n=3 experiments; significance (**p < 0.01 and ***p < 0.001) was determined by using Student's t-test.

Supplementary Table 1. Metal-free organic small molecules with visible-light excited RTP[3-14].

Reference	Molecular structure	Phosphorescence quantum yield (Φ) & lifetime (τ)	Mechanism
Chem. Mater. **31**, 5584−5591 (2019)<br>10.1021/acs.chemmater.9b01304<br>Ref. [3]	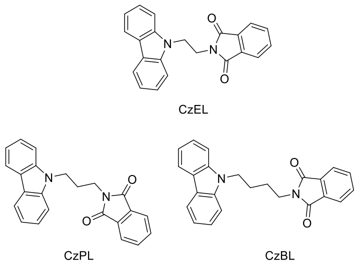	CzEL:<br>λ_{ex}: not mention, λ_{em} = 501 nm, τ = 762 ms<br><br>CzPL:<br>λ_{ex}: not mention, λ_{em} = 553 nm, τ = 605 ms<br><br>CzBL:<br>λ_{ex}: not mention, λ_{em} = 520 nm, τ = 222 ms	Not mention
J. Mater. Chem.C **7**, 9671-9677 (2019)<br>10.1039/C9TC03444G<br>Ref. [4]	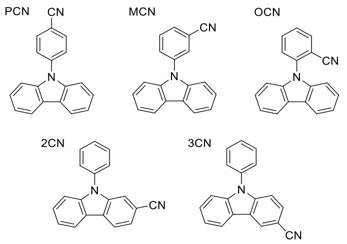	PCN:<br>λ_{ex} = 365 nm, λ_{em} = 546 nm, τ = 217.7 ms<br>λ_{ex} = 400 nm, λ_{em} = 546 nm, τ = 165.8 ms<br>λ_{ex} = 420 nm, λ_{em} = 546 nm, τ = 68.67 ms<br>λ_{ex} = 440 nm, λ_{em} = 546 nm, τ = 19.25 ms<br>λ_{ex} = 460 nm, λ_{em} = 546 nm, τ = 9.115 ms<br><br>MCN:<br>λ_{ex} = 365 nm, λ_{em} = 550 nm, τ = 261.9 ms<br>λ_{ex} = 400 nm, λ_{em} = 550 nm, τ = 128.7 ms<br>λ_{ex} = 420 nm, λ_{em} = 550 nm, τ = 16.43 ms<br>λ_{ex} = 440 nm, λ_{em} = 550 nm, τ =	Strong ISC process and direct $S_0 \rightarrow T_1$ absorption

		16.96 ms<br>λ_{ex} = 460 nm, λ_{em} = 550 nm, τ = 5.959 ms<br><br>OCN:<br>λ_{ex} = 365 nm, λ_{em} = 545 nm, τ = 83.4 ms<br>λ_{ex} = 400 nm, λ_{em} = 545 nm, τ = 43.7 ms<br>λ_{ex} = 420 nm, λ_{em} = 545 nm, τ = 8.14 ms<br>λ_{ex} = 440 nm, λ_{em} = 545 nm, τ = 7.83 ms<br>λ_{ex} = 460 nm, λ_{em} = 545 nm, τ = 4.73 ms<br><br>2CN:<br>λ_{ex} = 365 nm, λ_{em} = 520 nm, τ = 23.07 ms<br>λ_{ex} = 400 nm, λ_{em} = 520 nm, τ = 66.32 ms<br>λ_{ex} = 420 nm, λ_{em} = 520 nm, τ = 27.33 ms<br>λ_{ex} = 440 nm, λ_{em} = 520 nm, τ = 12.52 ms<br>λ_{ex} = 460 nm, λ_{em} = 520 nm, τ = 10.91 ms<br><br>3CN:<br>λ_{ex} = 365 nm, λ_{em} = 515 nm, τ = 346.7 ms<br>λ_{ex} = 400 nm, λ_{em} = 515 nm, τ = 110.9 ms<br>λ_{ex} = 420 nm, λ_{em} = 515 nm, τ = 118.2 ms<br>λ_{ex} = 440 nm, λ_{em} = 515 nm, τ = 102.0 ms<br>λ_{ex} = 460 nm, λ_{em} = 515 nm, τ = 29.65 ms	
Adv. Opt. Mater. **9**, 2101075 (2021) 10.1002/adom.202101075<br>Ref. [5]	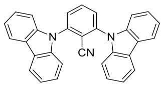<br>DCBN	Crystalline phase 1 :<br>λ_{ex} = 300 nm, Φ = 5.5%, λ_{em} = 544 nm, τ = 940 ms<br>λ_{ex} = 400 nm, Φ = 3.3%, λ_{em} = 544 nm, τ = 922 ms<br>λ_{ex} = 480 nm, Φ = 8.1%, λ_{em} = 544 nm, τ = 84 ms<br><br>Crystalline phase 2 :<br>λ_{ex} = 300 nm, Φ = 1.6%, λ_{em} = 546 nm, τ = 941 ms<br>λ_{ex} = 400 nm, Φ = 1.5%, λ_{em} = 546 nm, τ = 943 ms	Unclear

		λ_{ex} = 480 nm, Φ = 4.1%, λ_{em} = 546 nm, τ = 117 ms	
Adv. Mater. **29**, 1701244 (2017)<br>10.1002/adma.201701244<br>Ref. [6]	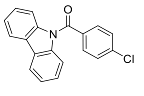<br>CPhCz	λ_{ex} = 410 nm, Φ = 8.2%, λ_{em} = 468 nm, τ = 824 ms; λ_{em} = 570 nm, τ = 847 ms; λ_{em} = 530 nm, τ = 836 ms	Intermolecular interaction
J. Am. Chem. Soc. **141,** 5045−5050 (2019)<br>10.1021/jacs.9b00859<br>Ref. [7]	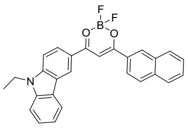<br>H-NpCzBF$_2$<br>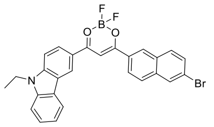<br>Br-NpCzBF$_2$<br>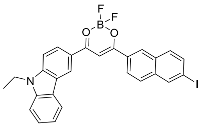<br>I-NpCzBF$_2$	H-NpCzBF:<br>λ_{ex} = 470 nm, λ_{em} = 640 nm, τ = 0.13 ms<br><br>Br-NpCzBF:<br>λ_{ex} = 470 nm, λ_{em} = 647 nm, τ = 0.58 ms<br><br>I-NpCzBF:<br>λ_{ex} = 470 nm, λ_{em} = 655 nm, τ = 0.56 ms	Dimer
Chem. Sci. **10**, 5031-5038, (2019)<br>10.1039/C8SC05198D<br>Ref. [8]	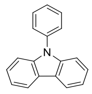<br>PhCz<br>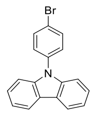<br>*p*BrPhCz<br>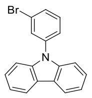<br>*m*BrPhCz<br>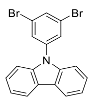<br>DBrPhCz	PhCz:<br>λ_{ex} = 400 nm, Φ = 1.8%, λ_{em} = 548 / 596 nm, τ = 0.27 / 0.26 s<br><br>pBrPhCz:<br>λ_{ex} = 400 nm, Φ = 9.5%, λ_{em} = 550 / 598 nm, τ = 0.25 / 0.23 s<br><br>mBrPhC:<br>λ_{ex} = 400 nm, Φ = 6.6%, λ_{em} = 548 / 598 nm, τ = 0.18 / 0.17 s<br><br>DBrPhCz:<br>λ_{ex} = 400 nm, Φ = 8.2%, λ_{em} = 549 / 597 nm, τ = 0.08 / 0.07	Efficient direct S0 → T1 absorption with the combined and synergetic effects of the heteroatom incorporation and internal and external heavy-atom interactions
Adv. Mater. **34**, 2201280 (2022)<br>10.1002/adma.202201280<br>Ref. [9]	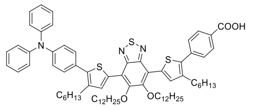<br>s-DTBT<br>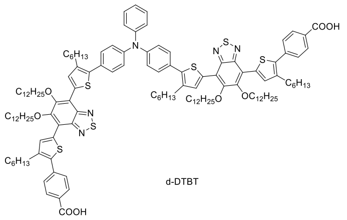<br>d-DTBT<br>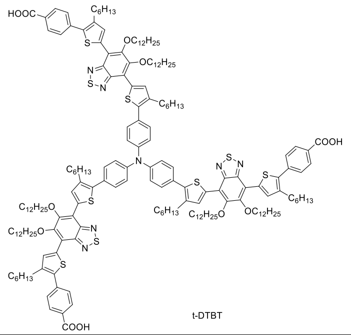<br>t-DTBT	s-DTBT:<br>λ_{ex}: not mention, λ_{em} = 635 nm, τ = 344 ms<br><br>d-DTBT:<br>λ_{ex}: not mention, λ_{em} = 640 nm, τ = 285 ms<br><br>t-DTBT:<br>λ_{ex}: not mention, λ_{em} = 660 nm, τ = 296 ms	Various electronic pull–push systems

ACS Appl. Mater. Interfaces **11**, 18103−18110 (2019) 10.1021/acsami.9b01615 Ref. [10]	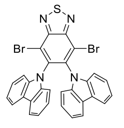 DBCz-BT	λ_{ex} = 400 nm, λ_{em} = 602 nm, τ = 504.6 us	Not mention
Dyes Pigm. **188**, 109178 (2021) 10.1016/j.dyepig.2021.109178 Ref. [11]	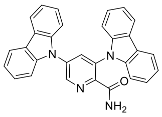	λ_{ex} = 365 nm, λ_{em} = 468 nm, τ = 295.78 ms; λ_{em} = 503 nm, τ = 338.99 ms; λ_{em} = 547 nm, τ = 342.40 ms; λ_{em} = 561 nm, τ = 270.82 ms; λ_{em} = 594 nm, τ = 338.76 ms	Not mention
J. Mater. Chem. C **8**, 11603-11609 (2020) 10.1039/D0TC00738B Ref. [12]	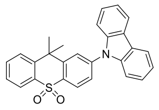 CzSO 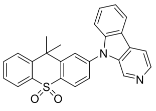 CbSO	CzSO: λ_{ex} = 440 nm, Φ = 26.3%, λ_{em} = 490 nm, τ = 0.49 s<br><br>CbSO: λ_{ex} = 440 nm, Φ = 4.4%, λ_{em} = 460 nm, τ = 0.44 s	Intermolecular through-space charge-transfer character
J. Phys. Chem. C **123**, 22104−22113 (2019) 10.1021/acs.jpcc.9b07762 Ref. [13]	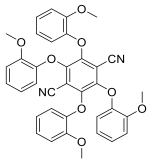	λ_{ex} = 350 nm, Φ = 0.58%, λ_{em} = 420 nm, τ = 24.85 ms; λ_{em} = 475 nm, τ = 39.00 ms; λ_{em} = 560 nm, τ = 46.14 ms	Not mention
ACS Appl. Mater. Interfaces **12**, 18385-18394 (2020) 10.1021/acsami.0c04005 Ref. [14]	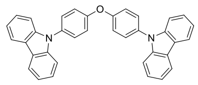 ODCz 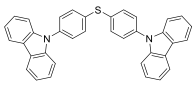 SDCz 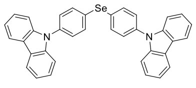 SeDCz 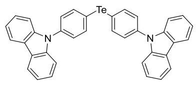 TeDCz	ODCz: λ_{ex}: not mention, Φ = 0.3%, λ_{em} = 570 nm, τ = 173 ms<br><br>SDCz: λ_{ex}: not mention, Φ = 1%, λ_{em} = 570 nm, τ = 318 ms,<br><br>SeDCz: λ_{ex}: not mention, Φ = 6.8%, λ_{em} = 572 nm, τ = 124 ms; λ_{em} = 618 nm, τ = 96 ms<br><br>TeDCz: λ_{ex}: not mention, Φ = 0.01%, λ_{em} = 571 nm, τ = 73 ms; λ_{em} = 623 nm, τ = 80 ms	Not mention

Supplementary Table 2. Lifetimes of TpPX at their corresponding peaks.

Compound	Atmosphere	λ (nm)<br>τ^a (ms) / τ^b (ms)				
TpPMe (crys.)		433	456	520	600	650
	air	140 / 140	210 / 0.15	180 / 140	410 / 390	510 / 460
	vac.[c]	170 / 140	190 / 0.15	230 / 160	420 / 400	510 / 490
TpPMe (amor.)				565	600	650
	air	-	-	2.6 / 0.60	3.3 / 1.9	4.6 / 5.3
	vac.	-	-	24 / 3.2	54 / 390	44 / 450
TpPBr (crys.)				510	610	645
	air	-	-	68 / -	230 / 190	210 / 200
	vac.	-	-	390 / -	220 / 210	230 / 210
TpPBr (amor.)				510	602/610	645
	air	-	-	0.58 / -	2.0 / 1.6	1.9 / 1.7
	vac.	-	-	1.5 / -	120 / 230	110 / 210
TpPI (crys.)					608	655
	air	-	-	-	62 / 72	59 / 71
	vac.	-	-	-	66 / 70	53 / 65
TpPI (amor.)					608	655
	air	-	-	-	2.9 / 31	3.1 / 120
	vac.	-	-	-	50 / 130	47 / 140
TpP (crys.)					610	645
	air	-	-	-	530 / 390	570 / 450
	vac.	-	-	-	550 / 350	610 / 460
TpP (amor.)					610	645
	air	-	-	-	4.1 / 1.5	4.8 / 1.4
	vac.	-	-	-	28 / 24	31 / 20
TpPF (crys.)		465	496		603	650
	air	140 / -	140 / -	-	480 / 470	460 / 440
	vac.	160 / -	150 / -	-	61 / 95	600 / 620
TpPF (amor.)		465	496		603	650
	air	0.33 / -	0.50 / -	-	2.2 / 3.4	0.95 / 2.3
	vac.	0.41 / -	0.46 / -	-	61 / 95	- / 280
TpPOMe (crys.)					572	630
	air	-	-	-	31 / 26	44 / 31
	vac.	-	-	-	35 / 35	52 / 41
TpPOMe (amor.)					572	630
	air	-	-	-	0.65 / 1.0	0.41 / 0.69
	vac.	-	-	-	2.5 / 6.2	9.7 / 8.4

[a] λ_{ex} = 340 nm

[b] λ_{ex} = 415 nm

[c] in a vacuum.

Supplementary Table 3. Photophysical parameters of Tp and TpPX powder at room temperature in air.

Chromophore	Φ_F (%)[a]	Φ_{ISC} (%)[b]	λ_F (nm)	τ_F (ns)[a]	k_F (10^7 s^{-1})[c]	k_{ISC} (10^7 s^{-1})[d]	Φ_P (%)[e]	λ_P (nm)	τ_P (ms)[f]	k_P (s^{-1})[g]
Tp	34.96	65.04	425	9.54	3.664	6.818	2.18	590	1147	0.029
TpP	14.40	85.60	480	12.29	1.171	6.963	10.25	610	390	0.307
TpPF	15.28	84.72	463	6.33	2.413	13.378	4.25	603	470	0.107
TpPBr	6.24	93.76	537	5.60	1.115	16.742	6.71	610	190	0.376
TpPI	0.62	99.38	500	2.46	0.252	40.328	0.76	608	72	0.106
TpPMe	14.82	85.18	530	6.25	2.369	13.621	9.26	600	390	0.279
TpPOMe	1.80	98.20	500	8.59	0.209	11.435	1.93	630	26	0.756

[a] λ_{ex} = 405 nm;

[b] Considering $k_F \ggg k_{IC}$ (RT), k_{ISC} (RT) $\ggg k_{IC}$ (RT), it can be approximated as $\Phi_{ISC} = 1-\Phi_F$;

[c] Calculated $k_F = \Phi_F/\tau_F$;

[d] Calculated $k_{ISC} = \Phi_{ISC}/\tau_F$;

[e] Calculated from aggregates by peak fitting;

[f] λ_{ex} = 415 nm;

[g] Calculated $k_P = \Phi_P \times \Phi_{ISC}^{-1} \times \tau_P^{-1}$.

Supplementary Table 4. Energy level of monomer and aggregate of compounds.

Compound	Monomer[a]			Aggregate[b]		
	S_1 (eV)	T_1 (eV)	ΔE_{ST} (eV)	S_1*(eV)	T_1*(eV)	ΔE_{ST} (eV)
**TpP**	3.092	2.696	0.40	2.767	2.033	0.73
**TpPF**	3.348	2.689	0.66	2.793	2.056	0.74
**TpPBr**	3.204	2.684	0.52	2.758	2.033	0.73
**TpPI**	3.207	2.696	0.51	2.864	2.039	0.82
**TpPMe**	3.017	2.696	0.32	2.856	2.067	0.79
**TpPOMe**	3.221	2.725	0.50	2.756	2.163	0.59

[a] The first excited singlet state (S_1) and the first excited triplet state (T_1) of monomer were calculated from prompt luminescence spectra of the compound in solution at room temperature and delayed luminescence spectra at 77 K, respectively.

[b] The S_1* and T_1* states of aggregation were calculated from prompt luminescence spectra and delayed luminescence spectra of the compound powder at room temperature.

Supplementary Table 5. Lifetimes of TpPBr powder at the 605 nm peak at temperatures from 77 to 300 K in a vacuum (λ_{ex} = 340 nm).

Temperature (K)	τ (s) at λ_{em} = 605 nm (A)		
77	1.72 (45%)	0.55 (30%)	0.20 (25%)
100	1.69 (50%)	0.61 (39%)	0.16 (11%)
150	1.69 (52%)	0.50 (37%)	0.13 (11%)
200	1.63 (53%)	0.51 (36%)	0.12 (11%)
250	1.29 (56%)	0.47 (33%)	0.13 (11%)
300	0.12 (65%)	0.42 (35%)	-

Supplementary Table 6. Crystal data and structure refinement for TpPX[a].

Compound	Tp	TpP	TpPF	TpPBr	TpPI	TpPMe	TpPOMe
Empirical formula	$C_{18}H_{12}$	$C_{25}H_{16}O$	$C_{25}H_{15}FO$	$C_{25}H_{15}BrO$	$C_{25}H_{15}IO$	$C_{26}H_{18}O$	$C_{26}H_{18}O_2$
Formula weight (g mol^{-1})	228.28	332.38	350.37	411.28	458.27	346.40	362.40
Temperature (K)	292.99(10)	150.1(3)	150.0(5)	297.98(10)	298.0	149.99(10)	150.00
Crystal system	orthorhombic	monoclinic	monoclinic	triclinic	orthorhombic	triclinic	triclinic
Space group	$P2_12_12_1$	$P2_1/c$	$P2_1/c$	P-1	$Pca2_1$	P-1	P1
a (Å)	5.2736(2)	11.3303(2)	11.3494(3)	7.8805(5)	29.089(2)	7.63400(10)	4.009(3)
b (Å)	13.1539(5)	11.4559(2)	11.1405(3)	9.1092(4)	4.4826(3)	9.0718(2)	11.307(9)
c (Å)	16.7493(7)	12.9004(2)	13.3658(3)	13.0214(6)	28.013(2)	13.1331(2)	20.002(17)
α (°)	90	90	90	71.440(4)	90	69.979(2)	94.25(3)
β (°)	90	94.587(2)	91.592(2)	82.170(5)	90	81.373(2)	95.52(3)
γ (°)	90	90	90	86.136(4)	90	85.787(2)	99.97(3)
Volume (Å^3)	1161.87(8)	1669.09(5)	1689.29(7)	877.58(8)	3652.7(5)	844.65(3)	885.0(12)
Z	4	4	4	2	8	2	2
ρ_{calc} (g cm^{-3})	1.305	1.323	1.378	1.556	1.667	1.362	1.360
μ (mm^{-1})	0.560	0.614	0.724	3.273	9.277	0.628	0.425
F (000)	480.0	696.0	728.0	416.0	1808.0	364.0	380.0
Radiation	CuKα (λ = 1.54184)	CuKα (λ = 1.54184)	CuKα (λ = 1.54184)	CuKα (λ = 1.54184)	CuKα (λ = 1.34138)	CuKα (λ = 1.54184)	GaKα (λ = 1.34138)
2Θ range for data collection (°)	8.548 to 145.822	7.828 to 145.69	7.792 to 145.952	7.214 to 145.584	5.286 to 105.962	7.228 to 145.762	3.878 to 104.186
Index ranges	$-6 \leqslant h \leqslant 4, -16 \leqslant k \leqslant 15, -19 \leqslant l \leqslant 20$	$-13 \leqslant h \leqslant 12, -8 \leqslant k \leqslant 14, -15 \leqslant l \leqslant 10$	$-13 \leqslant h \leqslant 13, -13 \leqslant k \leqslant 13, -16 \leqslant l \leqslant 14$	$-9 \leqslant h \leqslant 9, -10 \leqslant k \leqslant 11, -15 \leqslant l \leqslant 15$	$-34 \leqslant h \leqslant 34, -5 \leqslant k \leqslant 5, -33 \leqslant l \leqslant 33$	$-9 \leqslant h \leqslant 8, -11 \leqslant k \leqslant 11, -16 \leqslant l \leqslant 16$	$-4 \leqslant h \leqslant 4, -13 \leqslant k \leqslant 13, -23 \leqslant l \leqslant 23$
Reflections collected	5885	6169	18146	5751	108236	28931	5999
Independent reflections	2190 [R_{int} = 0.1311, R_{sigma} = 0.0850]	3231 [R_{int} = 0.0253, R_{sigma} = 0.0269]	3335 [R_{int} = 0.0388, R_{sigma} = 0.0231]	3389 [R_{int} = 0.0431, R_{sigma} = 0.0441]	6370 [R_{int} = 0.0671, R_{sigma} = 0.0263]	3329 [R_{int} = 0.0321, R_{sigma} = 0.0141]	5999 [R_{int} = merged, R_{sigma} = 0.0748]
Data/restraints/parameters	2190/0/163	3231/0/235	3335/0/244	3389/0/244	6370/1/476	3329/0/245	5999/3/413
Goodness-of-fit on F^2	1.093	1.044	0.983	1.257	1.120	1.038	1.085
Final *R* indexes [I>=2σ (I)]	R_1 = 0.1039, wR_2 = 0.2537	R_1 = 0.0452, wR_2 = 0.1246	R_1 = 0.0476, wR_2 = 0.1333	R_1 = 0.0525, wR_2 = 0.1709	R_1 = 0.0828, wR_2 = 0.2251	R_1 = 0.0417, wR_2 = 0.1168	R_1 = 0.0908, wR_2 = 0.2505
Final R indexes [all data]	R_1 = 0.1199, wR_2 = 0.2915	R_1 = 0.0492, wR_2 = 0.1291	R_1 = 0.0547, wR_2 = 0.1392	R_1 = 0.0647, wR_2 = 0.1762	R_1 = 0.0867, wR_2 = 0.2335	R_1 = 0.0426, wR_2 = 0.1179	R_1 = 0.1066, wR_2 = 0.2674
Largest diff. peak/hole (e Å^{-3})	0.41/-0.39	0.39/-0.20	0.47/-0.21	0.48/-0.68	0.50/-1.16	0.31/-0.36	0.44/-0.38

[a] The obtained crystal structures have been deposited at the Cambridge Crystallographic Data Centre and allocated the deposition numbers: 2276028 (TpP), 2276075 (TpPF), 2276105 (TpPBr), 2276126 (TpPI), 2276143 (TpPMe), (2304903) TpPOMe.

Supplementary Table 7. Schematic representation of aggregation models. d_1 represents vertical distance between two adjacent triphenylene planes while d_2 was the centroid distance of two adjacent triphenylene. The angle between the transition dipoles and the interconnected axis is indicated by θ.

Compound-dimer	d_1 (Å)	d_2 (Å)	θ	transition electric dipole moments of S_0-S_1 (a.u.)	H/J-aggregation
TpP	3.390	3.613	43.8	(0.1271, -0.0780, -0.0935)	J
TpPF	3.427	3.631	16.4	(-0.1434, -0.0841, 0.0960)	J
TpPBr	3.695	4.496	39.0	(-0.0498, -0.0752, -0.0994)	J
	3.554	3.823	76.9	(-0.0498, -0.0752, -0.0994)	H
TpPMe	3.590	4.823	34.3	(0.0414, 0.0642, 0.0954)	J
	3.472	3.748	68.7	(0.0414, 0.0642, 0.0954)	H
TpPI	3.399	4.483	66.4	(-0.0153, 0.0761, 0.1739)	H
	3.481	4.483	71.8	(0.0829, -0.0286, -0.0261)	H
TpPOMe	3.380	4.009	64.0	(-0.0829, 0.1141, -0.1253)	H
	3.480	4.564	66.0	(0.0736, -0.1177, -0.1160)	H
Tp	3.374	5.274	65.1	(0.0025, 0.0052, -0.0014)	H

Supplementary Table 8. Comparison of room-temperature phosphorescence and corresponding lifetimes of pure organic nanoparticles reported in our work and literature since 2016. The chromophores in these nanoparticles are single-component[6,7,9,10,14-41].

Supplementary reference	Phosphorescence quantum yield (Φ) & lifetime (τ)
Adv. Mater. **29**, 1701244 (2017)<br>10.1002/adma.201701244<br>Ref. [6]	λ_{em} = 530 nm, τ = 0.693 s;<br>λ_{em} = 570 nm, τ = 0.655 s
Adv. Mater. **34**, 2201280 (2022)<br>10.1002/adma.202201280<br>Ref. [9]	λ_{em} = 650 nm, τ = 7.17 us;<br>λ_{em} = 667 nm, τ = 4.19 us;<br>λ_{em} = 624 nm, τ = 3.97 us
ACS Appl. Mater. Interfaces **12**, 18385-18394 (2020)<br>10.1021/acsami.0c04005<br>Ref. [14]	λ_{em} = 580 nm, τ = 34 ms
ACS Appl. Mater. Interfaces **11**, 18103-18110 (2019)<br>10.1021/acsami.9b01615<br>Ref. [10]	λ_{em} = 602 nm, τ = 203.1 us
Nat. Commun. **13**, 186 (2022)<br>10.1038/s41467-021-27914-0<br>Ref. [16]	Φ = 3.1%, λ_{em} = 681/732 nm, τ = 70 ms
J. Am. Chem. Soc. **141**, 5045-5050 (2019)<br>10.1021/jacs.9b00859<br>Ref. [7]	λ_{em} = 636 nm, τ = 27.6 us;<br>λ_{em} = 625 nm, τ = 29.0 us;<br>λ_{em} = 628 nm, τ = 17.2 us
Polym. Chem. **14**, 318-323 (2023)<br>10.1039/D2PY00937D<br>Ref. [17]	λ_{em} = 520 nm, τ = 2.49 ms
Chin. J. Chem. **41**, 1575-1582 (2023)<br>10.1002/cjoc.202200838<br>Ref. [18]	λ_{em} = 635 nm, τ: not mention
Sci. China Chem. **66**, 816-825 (2023)<br>10.1007/s11426-022-1469-2<br>Ref. [19]	λ_{em} = 476 nm, τ = 178.3 ms
Dyes Pigm. **193**, 109520 (2021)<br>10.1016/j.dyepig.2021.109520<br>Ref. [20]	λ_{em} = 656 nm, τ = 27.6 us

Nat. Commun. **12**, 4883 (2021)<br>10.1038/s41467-021-25174-6<br>Ref. [21]	Φ = 3.5%, λ_{em} = 537 nm, τ = 8.5 us;<br>Φ = 3.6%, λ_{em} = 549 nm, τ = 7.4 us;
Chem. Sci. **12**, 1851-1857 (2021)<br>10.1039/D0SC05343K<br>Ref. [22]	λ_{em} = 580 nm, τ = 289 us
Anal. Chem. **93**, 6516-6522 (2021)<br>10.1021/acs.analchem.1c00423<br>Ref. [23]	Φ = 7.6%, λ_{em} = 550 nm, τ = 284.59 ms; λ_{em} = 590 nm, τ = 264.95 ms
Nat. Mater. **8**, 747-751 (2009)<br>10.1038/nmat2509<br>Ref. [24]	λ_{em} = 528 nm, τ = 4.82 ms
10.26434/chemrxiv-2022-z59ds<br>Ref. [25]	Φ = 22.9%, λ_{em} = 500 nm, τ = 49 ms
Sci. China Mater. **63,** 316-324 (2020)<br>10.1007/s40843-019-1191-9<br>Ref. [26]	λ_{em} = 600 nm, τ = 167 us
CCS Chem. **4**, 2550–2559 (2022)<br>10.31635/ccschem.021.202101120<br>Ref. [27]	λ_{em} = 606 nm, τ = 139 ms
Angew. Chem. Int. Ed. **60**, 18630 (2021)<br>10.1002/anie.202103965<br>Ref. [28]	Φ = 26%, λ_{em} = 731 nm, τ = 0.94 us;<br>Φ = 21%, λ_{em} = 764 nm, τ = 1.8 us
Biosens. Bioelectron. **199**, 113889 (2022)<br>10.1016/j.bios.2021.113889.<br>Ref. [29]	Φ = 6.13 ± 2.3%, λ_{em} = 525 nm, τ = 1.0 ms
ChemPlusChem **82**, 399 (2017)<br>10.1002/cplu.201600520<br>Ref. [30]	λ_{em} = 545 nm, τ = 62 ms;<br>λ_{em} = 589 nm, τ = 4.1 ms;<br>λ_{em} = 600 nm, τ = 1.6 ms;
Adv. Sci. **9**, 2200524 (2022)<br>10.1002/advs.202200524<br>Ref. [31]	λ_{em} = 540 nm, τ = 1.80 ms;<br>λ_{em} = 635 nm, τ = 83.8 us
Adv.Mater. ***29***, 1606665 *(***2017)**<br>10.1002/adma.201606665<br>Ref. [32]	Φ = 11%, λ_{em} = 530 nm, τ = 861 ms
Angew. Chem. Int. Ed. **59**, 9928 (2020)<br>10.1002/anie.201914513<br>Ref. [15]	λ_{em} = 565 nm, τ = 0.190 ms
Nat. Commun. **11**, 4655 (2020)<br>10.1038/s41467-020-18520-7<br>Ref. [33]	Φ = 7.58%, λ_{em} = 500 nm, τ = 4.33 ms
Angew. Chem. Int. Ed. **53**, 14149 (2014)<br>10.1002/anie.201407402<br>Ref. [34]	λ_{em} = 552 nm, τ = 0.56 ms
Angew. Chem. Int. Ed. **56**, 12160 (2017)<br>10.1002/anie.201705945<br>Ref. [35]	λ_{em} = 570 nm, τ = 140 ms
J. Am. Chem. Soc. **139**, 14792 (2017)<br>10.1021/jacs.7b08710<br>Ref. [36]	Φ = 34%, λ_{em} = 520 nm, τ = 232 ms
Adv. Funct. Mater. **30**, 1907282 (2020)<br>10.1002/adfm.201907282<br>Ref. [37]	Φ = 26.3%, λ_{em} = 515 nm, τ = 0.27 ms;<br>Φ = 19.4%, λ_{em} = 607 nm, τ = 3.30 ms;<br>Φ = 7.0%, λ_{em} = 605 nm, τ = 1.20 ms
Mater. Horizons **6**, 1259 (2019)<br>10.1039/C9MH00220K<br>Ref. [38]	λ_{em} = 544 nm, τ = 232 ms
ACS Nano **2**, 1252 (2008)<br>10.1021/nn7003525	λ_{em} = 509 nm, τ = 200 ms

Ref. [39]	
ACS Sens. **1**, 1366 (2016)<br>10.1021/acssensors.6b00533<br>Ref. [40]	λ_{em} = 543 nm, τ = 127 ms;<br>λ_{em} = 559 nm, τ = 12.3 ms;<br>λ_{em} = 565 nm, τ = 1.7 ms
J. Mater. Chem. C **7**, 9917 (2019)<br>10.1039/C9TC02266J<br>Ref. [41]	Φ = 18.8%, λ_{em} = 460 nm, τ = 0.126 ms;<br>Φ = 21.8%, λ_{em} = 570 nm, τ = 0.033 ms
**This work**	**Φ = 1%**<br>**λ_{em} = 510 nm, τ = 0.10 s;**<br>**λ_{em} = 610 nm, τ = 0.23 s;**<br>**λ_{em} = 645 nm, τ =0.29 s**

Supplementary references

1. Liu, Z., Lu, T. & Chen, Q. An sp-hybridized all-carboatomic ring, cyclo[18]carbon: electronic structure, electronic spectrum, and optical nonlinearity. *Carbon* **165**, 461-467 (2020).
2. Lu, T. et al. Interaction region indicator (IRI): a very simple real space function clearly revealing both chemical bonds and weak interactions. *Chemistry-Methods* **1**, 231-239 (2021).
3. Wang, X. et at. Multicolor ultralong organic phosphorescence through alkyl engineering for 4D coding applications. *Chem. Mater.* **31**, 5584−5591 (2019).
4. Wang, Y. et al. Cyanophenylcarbazole isomers exhibiting different UV and visible light excitable room temperature phosphorescence. *J. Mater. Chem. C* **7**, 9671-9677 (2019).
5. Wang, Y. et al. Persistent organic white-emitting afterglow from ultralong thermally activated delayed fluorescence and room-temperature phosphorescence. *Adv. Opt. Mater.* **9**, 2101075 (2021).
6. Cai, S. et al. Visible-light-excited ultralong organic phosphorescence by manipulating intermolecular interactions. *Adv. Mater.* **29**, 1701244 (2017).
7. Wang, X.F. et al. Pure organic room temperature phosphorescence from excited dimers in self-assembled nanoparticles under visible and near-infrared irradiation in water. *J. Am. Chem. Soc.* **141**, 5045−5050 (2019).
8. Yuan, J. et al. Direct population of triplet excited states through singlet–triplet transition for visible-light excitable organic afterglow. *Chem. Sci.* **10**, 5031-5038 (2019).
9. Fan, Y. et al. Mobile phone flashlight-excited red afterglow bioimaging. *Adv. Mater.* **34**, 2201280 (2022).
10. Shi, H. et al. A Highly efficient red metal-free organic phosphor for time-resolved luminescence imaging and photodynamic therapy. *ACS Appl. Mater. Interfaces* **11**, 18103−18110 (2019).
11. Liu, J. et al. Crystal-state quad-mode triplet emissions of D-A-A’-D type phosphors with AIEE and visible-light-excited persistent phosphorescence. *Dyes Pigm.* **188**, 109178 (2021).
12. Luo, Y. et al. Ultralong organic luminogens with color-tunability via intermolecular through-space charge-transfer characters. *J. Mater. Chem. C* **8**, 11603-11609 (2020).
13. Bhatia, H. & Ray, D. Use of dimeric excited states of the donors in D4-A systems for accessing white light emission, afterglow, and invisible security ink. *J. Phys. Chem. C* **123**, 22104−22113 (2019).
14. Xu, L. et al. Ultralong organic phosphorescent nanocrystals with long-lived triplet excited states for afterglow imaging and photodynamic therapy. *ACS Appl. Mater. Interfaces* **12**, 18385-18394 (2020).
15. Wang, J. et al. Visible-light-excited room-temperature phosphorescence in water by cucurbit[8]uril-mediated supramolecular assembly. *Angew. Chem. Int. Ed.* **59**, 9928-9933 (2020).
16. Xiao, F. et al. Guest-host doped strategy for constructing ultralong-lifetime near-infrared organic phosphorescence materials for bioimaging. *Nat. Commun.* **13**, 186 (2022).

17. Xu, W. et al. Fabrication of nano-objects with morphology-correlated room-temperature phosphorescence and their application in information encryption. *Polym. Chem.* **14**, 318-323 (2023).
18. Si, Y. et al. Organic host-guest materials with bright red room-temperature phosphorescence for persistent bioimaging. *Chinese J. Chem.* **41**, 1575-1582 (2023).
19. Tu, L. et al. How temperature and hydrostatic pressure impact organic room temperature phosphorescence from H-aggregation of planar triarylboranes and the application in bioimaging. *Sci. Chi. Chem.* **66**, 816-825 (2023).
20. Lv, A. et al. Molecular conformation dependence of phosphorescence lifetime in organic aggregates. *Dyes Pigm.* **193**, 109520 (2021).
21. Yang, J. et al. Rational design of pyrrole derivatives with aggregation-induced phosphorescence characteristics for time-resolved and two-photon luminescence imaging. *Nat. Commun.* **12**, 4883 (2021).
22. Shen, F.F. et al. Purely organic light-harvesting phosphorescence energy transfer by beta-cyclodextrin pseudorotaxane for mitochondria targeted imaging. *Chem. Sci.* **12**, 1851-1857 (2020).
23. Zhou, Y. et al. Microscopic afterglow bioimaging by ultralong organic phosphorescent nanoparticles in living cells and zebrafish. *Anal. Chem.* **93**, 6516-6522 (2021).
24. Zhang, G. et al. A dual-emissive-materials design concept enables tumour hypoxia imaging. *Nat. Mater.* **8**, 747-751 (2009).
25. Li, Z. et al. Lighting up early metastases by phosphorescence imaging. 10.26434/chemrxiv-2022-z59ds
26. Wang, S. et al. Biocompatible metal-free organic phosphorescent nanoparticles for efficiently multidrug-resistant bacteria eradication. *Sci. China Mater*. **63**, 316-324 (2019).
27. Dai, W. et al. Red-emissive organic room-temperature phosphorescence material for time-resolved luminescence bioimaging. *CCS Chemistry* **4**, 2550-2559 (2022).
28. Paisley, N.R. et al. Near-infrared-emitting boron-difluoride-curcuminoid-based polymers exhibiting thermally activated delayed fluorescence as biological imaging probes. *Angew. Chem. Int. Ed.* **60**,18630-18638 (2021).
29. Kang, D.H. et al. Highly sensitive and quantitative biodetection with lipid-polymer hybrid nanoparticles having organic room-temperature phosphorescence. *Biosens. Bioelectron.* **199**, 113889 (2022).
30. DeRosa, C.A. et al. Oxygen-sensing difluoroboron thienyl phenyl beta-diketonate polylactides. *Chempluschem* **82**, 399-406 (2017).
31. Dai, X.Y. et al. A highly efficient phosphorescence/fluorescence supramolecular switch based on a bromoisoquinoline cascaded assembly in aqueous solution. *Adv. Sci.* **9**, e2200524 (2022).
32. Zhen, X. et al. Ultralong phosphorescence of water-soluble organic nanoparticles for in vivo afterglow imaging. *Adv. Mater.* **29**, 1606665 (2017).
33. Zhou, W.L. et al. Ultralong purely organic aqueous phosphorescence supramolecular polymer for targeted tumor cell imaging. *Nat. Commun.* **11**, 4655 (2020).
34. Chen, H. et al. A rapidly self-healing supramolecular polymer hydrogel with photostimulated room-temperature phosphorescence responsiveness. *Angew. Chem. Int. Ed.* **53**, 14149-14152 (2014).
35. Fateminia, S.M.A. et al. Organic nanocrystals with bright red persistent room-temperature phosphorescence for biological applications. *Angew. Chem. Int. Ed.* **56**, 12160-12164 (2017).
36. Nicol, A. et al. Ultrafast delivery of aggregation-induced emission nanoparticles and pure organic phosphorescent nanocrystals by saponin encapsulation. *J. Am. Chem. Soc.* **139**, 14792-14799 (2017).
37. Wang, X.F. et al. Pure organic room temperature phosphorescence from unique micelle-assisted assembly of nanocrystals in water. *Adv. Funct. Mater.* **30**, 1907282 (2020).
38. Yuan, J. et al. Invoking ultralong room temperature phosphorescence of purely organic compounds through H-aggregation engineering. *Mater. Horizons* **6**, 1259-1264 (2019).
39. Pfister, A. et al. Boron polylactide nanoparticles exhibiting fluorescence and phosphorescence in aqueous medium. *ACS Nano*, **2**, 1252–1258 (2008).

40. DeRosa, C.A. et al. Oxygen sensing difluoroboron beta-diketonate polylactide materials with tunable dynamic ranges for wound imaging. *ACS Sens.* **1**, 1366-1373 (2016).

41. Wang, T. et al. A combinatory approach towards the design of organic polymer luminescent materials. *J. Mater. Chem. C*. **7**, 9917-9925 (2019)
