# Supplementary material for: Visible-light-excited robust room-temperature phosphorescence of dimeric single-component luminophores in the amorphous state
Source: Nat Commun. 2024 Apr 27;15:3598. doi: 10.1038/s41467-024-47937-7 (PMC11055858; doi:10.1038/s41467-024-47937-7)
Supplement: Supplementary file 3 — Description of Additional Supplementary Files [file 41467_2024_47937_MOESM3_ESM.pdf]

## Description of Additional Supplementary Files

File Name: Supplementary Movie 1
Description: Amorphous TpPBr powder (298 K, in air, $\lambda_{ex}$ = 405 nm)

File Name: Supplementary Movie 2
Description: Amorphous TpPBr powder (298 K, in air, iphone7 plus)

File Name: Supplementary Movie 3
Description: Amorphous TpPI powder (298 K, in air, $\lambda_{ex}$ = 405 nm)

File Name: Supplementary Movie 4
Description: TpPBr@F127 nanoparticles (2.44 mM, 298 K, in air, $\lambda_{ex}$ = 405 nm)
