# Supplementary material for: Visible-light-excited robust room-temperature phosphorescence of dimeric single-component luminophores in the amorphous state
Source: Nat Commun. 2024 Apr 27;15:3598. doi: 10.1038/s41467-024-47937-7 (PMC11055858; doi:10.1038/s41467-024-47937-7)
Supplement: Supplementary file 8 — Reporting Summary [file 41467_2024_47937_MOESM8_ESM.pdf]

nature portfolio

Corresponding author(s): Zhiyong Yang

Last updated by author(s): Mar 18, 2024

Reporting Summary

Nature Portfolio wishes to improve the reproducibility of the work that we publish. This form provides structure for consistency and transparency in reporting. For further information on Nature Portfolio policies, see our Editorial Policies and the Editorial Policy Checklist.

Statistics

For all statistical analyses, confirm that the following items are present in the figure legend, table legend, main text, or Methods section.

n/a	Confirmed	
☒	☐	The exact sample size (*n*) for each experimental group/condition, given as a discrete number and unit of measurement
☒	☐	A statement on whether measurements were taken from distinct samples or whether the same sample was measured repeatedly
☒	☐	The statistical test(s) used AND whether they are one- or two-sided<br>*Only common tests should be described solely by name; describe more complex techniques in the Methods section.*
☐	☒	A description of all covariates tested
☒	☐	A description of any assumptions or corrections, such as tests of normality and adjustment for multiple comparisons
☐	☒	A full description of the statistical parameters including central tendency (e.g. means) or other basic estimates (e.g. regression coefficient) AND variation (e.g. standard deviation) or associated estimates of uncertainty (e.g. confidence intervals)
☒	☐	For null hypothesis testing, the test statistic (e.g. *F*, *t*, *r*) with confidence intervals, effect sizes, degrees of freedom and *P* value noted<br>*Give P values as exact values whenever suitable.*
☒	☐	For Bayesian analysis, information on the choice of priors and Markov chain Monte Carlo settings
☒	☐	For hierarchical and complex designs, identification of the appropriate level for tests and full reporting of outcomes
☒	☐	Estimates of effect sizes (e.g. Cohen's *d*, Pearson's *r*), indicating how they were calculated

Our web collection on statistics for biologists contains articles on many of the points above.

Software and code

Policy information about availability of computer code

Data collection

1. Topspin 3.6 ICONNMR for 1H NMR and 13C NMR.
2. Standard Measurement for XRD detected on a RIGAKU X-ray powder diffraction (D-max 2200 VPC)
3. DSC 204 F1 Phoenix for DSC carried out on a differential scanning calorimeter (Netzsch DSC 204 F1)
4. UV Solutions and UVProbe2.43 for Uv-vis absorbance of the solution performed on Hitachi U-3900 spectrophotometer and powder obtained from Shimadzu UV-3600, respectively.
5. Fluoracle ® software for all measurements in Edinburgh FLS1000 steady/transient state fluorescence spectrometer, such as time-resolved emission spectroscopy, fluorescence intensity decay profile, prompt luminescence spectrum of powder, delayed luminescence spectrum with a delay time of 5-99 ms and PLQY.
6. DataStation v2.7 for photoluminescence (PL) decay profiles and Excitation-phosphorescence mapping performed on Horiba JY FL-3 steady-state/transient combined fluorescence spectrometer.
7. Spectrasuite beta for delayed luminescence spectrum performed on Ocean Optics QE65PRO of powder, solution and nanoparticles.
8. RFPC for prompt luminescence spectrum performed on fluorescence spectrometer (Shimadzu RF-5301PC) of the solution.
9. Particle Solution for DLS and Zeta using a Brookhaven EliteSizer.
10. FV3000 for confocal laser scanning microscopy images obtained from Olympus FV3000.
11. IndiGo for afterglow photographs of the living mice captured on IVIS Spectrum.
12. Afterglow photographs were captured by iphone12 camera with recording parameters of 1080p HD/60 fps or digital camera (Canon EOS 750D).

Data analysis

1. Mercury4.1.0 was used to analyze the packing mode of single crystals.
2. Jade was used to analyze XRD results.

3. The structures were solved by direct methods following the difference Fourier syntheses, and refined against all data using the SHELXTL software package as implemented in Olex2.
4. NETZSCH-Proteus Thermal Analysis was used to analyze DSC results.
5. Fluoracle ® software was used to analyze photophysical measurements performed on Edinburgh FLS1000 steady/transient state fluorescence spectrometer.
6. DAS6. V6.8 was used to analyze photophysical measurements performed on Horiba JY FL-3 steady-state/transient combined fluorescence spectrometer.
7. Particle Solution was used to analyze DLS and Zeta results using a Brookhaven EliteSizer.
8. Digital micrography was used to analyze the morphology of nanoparticles.
9. FV3000 was used to analyze CLSM images.
10. IndiGo was used to analyze afterglow photographs of the living mice captured on IVIS Spectrum.
11. General data analysis were performed on Origin 2018.
12. Natural transition orbitals (NTOs), interaction region indicator (IRI) were obtained by Multiwfn and visualized using GaussView 5.0 and VMD.[1, 2]. Spin-orbit coupling matrix elements (SOC) were calculated at the TDDFT by orca based on the M062X TZVP basis set, the angle between transition moment of the monomer and the interconnection of the centres were obtained from GaussView 5.0.

For manuscripts utilizing custom algorithms or software that are central to the research but not yet described in published literature, software must be made available to editors and reviewers. We strongly encourage code deposition in a community repository (e.g. GitHub). See the Nature Portfolio guidelines for submitting code & software for further information.

Data

Policy information about availability of data

All manuscripts must include a data availability statement. This statement should provide the following information, where applicable:

- Accession codes, unique identifiers, or web links for publicly available datasets
- A description of any restrictions on data availability
- For clinical datasets or third party data, please ensure that the statement adheres to our policy

The authors declare that the data supporting the findings of this study are available within the article and its Supplementary Information. Extra data are available from the corresponding authors upon reasonable request.

Research involving human participants, their data, or biological material

Policy information about studies with human participants or human data. See also policy information about sex, gender (identity/presentation), and sexual orientation and race, ethnicity and racism.

Reporting on sex and gender	This information has not been collected.
Reporting on race, ethnicity, or other socially relevant groupings	See above.
Population characteristics	See above.
Recruitment	See above.
Ethics oversight	See above.

Note that full information on the approval of the study protocol must also be provided in the manuscript.

Field-specific reporting

Please select the one below that is the best fit for your research. If you are not sure, read the appropriate sections before making your selection.

☒ Life sciences ☐ Behavioural & social sciences ☐ Ecological, evolutionary & environmental sciences

For a reference copy of the document with all sections, see nature.com/documents/nr-reporting-summary-flat.pdf

Life sciences study design

All studies must disclose on these points even when the disclosure is negative.

Sample size	No statistical method was used to predetermine the sample size for each study. In vivo and vitro studies, cells and living mice were repeated at least two times independently for evaluating the statistical significance.
Data exclusions	No data was excluded from this study.
Replication	All experiments were reproduced to reliably support conclusions stated in the manuscript. TEM and SAED experiment were repeated at least eight times with similar results. Confocal microscopy images were repeated at three times with similar results. Biological experiments were repeated at least three times.

Randomization	Cells and mice were randomly selected.
Blinding	The investigators were not blinded to allocation during experiments and outcome assessment since our data analyses are based on objectively measurable data.

Reporting for specific materials, systems and methods

We require information from authors about some types of materials, experimental systems and methods used in many studies. Here, indicate whether each material, system or method listed is relevant to your study. If you are not sure if a list item applies to your research, read the appropriate section before selecting a response.

Materials & experimental systems

n/a	Involved in the study
☒	☐ Antibodies
☐	☒ Eukaryotic cell lines
☒	☐ Palaeontology and archaeology
☐	☒ Animals and other organisms
☒	☐ Clinical data
☒	☐ Dual use research of concern
☒	☐ Plants

Methods

n/a	Involved in the study
☒	☐ ChIP-seq
☒	☐ Flow cytometry
☒	☐ MRI-based neuroimaging

Eukaryotic cell lines

Policy information about cell lines and Sex and Gender in Research

Cell line source(s)	Mouse prostate cancer RM-1 cells were provided by iCell.
Authentication	The cell line was authenticated by the supplier.
Mycoplasma contamination	Mycoplasma contamination have been tested.
Commonly misidentified lines (See ICLAC register)	None.

Animals and other research organisms

Policy information about studies involving animals; ARRIVE guidelines recommended for reporting animal research, and Sex and Gender in Research

Laboratory animals	BALB/c and C57BL/6 mice were purchased from the Laboratory Animal Center of Sun Yat-Sen University at the age of 4-6 weeks after birth.
Wild animals	None.
Reporting on sex	Mice were randomly selected without considering sex.
Field-collected samples	None.
Ethics oversight	Mice experiments were performed under a protocol approved by the Institutional Animal Care and Use Committee of Sun Yat-Sen University.

Note that full information on the approval of the study protocol must also be provided in the manuscript.

Plants

Seed stocks	None.
Novel plant genotypes	None.
Authentication	None.
